# Incomplete meiotic sex chromosome inactivation in the domestic dog

**DOI:** 10.1186/s12864-015-1501-9

**Published:** 2015-04-12

**Authors:** Federica Federici, Eskeatnaf Mulugeta, Sam Schoenmakers, Evelyne Wassenaar, Jos W Hoogerbrugge, Godfried W van der Heijden, Wiggert A van Cappellen, Johan A Slotman, Wilfred FJ van IJcken, Joop SE Laven, J Anton Grootegoed, Willy M Baarends

**Affiliations:** Department of Developmental Biology, Erasmus MC, University Medical Center, PO BOX 2040, 3000 CA Rotterdam, The Netherlands; Department of Obstetrics and Gynaecology, Erasmus MC, University Medical Center, PO BOX 2040, 3000 CA Rotterdam, The Netherlands; Department of Pathology, Erasmus Optical Imaging Centre, PO BOX 2040, 3000 CA Rotterdam, The Netherlands; Erasmus Center for Biomics, Erasmus MC, University Medical Center, Rotterdam, The Netherlands; Present address: Institut Curie, Genetics and Developmental Biology, Unit 11 et 13 rue Pierre et Marie Curie, 75248 Paris, Cedex 05 France

**Keywords:** Meiosis, Sex chromosomes, Spermatogenesis, Meiotic sex chromosome inactivation, Synaptonemal complex, Transcriptome, MSCI, Dog, Spermatocytes, Spermatids, DNA double-strand break repair

## Abstract

**Background:**

In mammalian meiotic prophase, homologous chromosome recognition is aided by formation and repair of programmed DNA double-strand breaks (DSBs). Subsequently, stable associations form through homologous chromosome synapsis. In male mouse meiosis, the largely heterologous X and Y chromosomes synapse only in their short pseudoautosomal regions (PARs), and DSBs persist along the unsynapsed non-homologous arms of these sex chromosomes. Asynapsis of these arms and the persistent DSBs then trigger transcriptional silencing through meiotic sex chromosome inactivation (MSCI), resulting in formation of the XY body. This inactive state is partially maintained in post-meiotic haploid spermatids (postmeiotic sex chromatin repression, PSCR). For the human, establishment of MSCI and PSCR have also been reported, but X-linked gene silencing appears to be more variable compared to mouse. To gain more insight into the regulation and significance of MSCI and PSCR among different eutherian species, we have performed a global analysis of XY pairing dynamics, DSB repair, MSCI and PSCR in the domestic dog (*Canis lupus familiaris*), for which the complete genome sequence has recently become available, allowing a thorough comparative analyses.

**Results:**

In addition to PAR synapsis between X and Y, we observed extensive self-synapsis of part of the dog X chromosome, and rapid loss of known markers of DSB repair from that part of the X. Sequencing of RNA from purified spermatocytes and spermatids revealed establishment of MSCI. However, the self-synapsing region of the X displayed higher X-linked gene expression compared to the unsynapsed area in spermatocytes, and was post-meiotically reactivated in spermatids. In contrast, genes in the PAR, which are expected to escape MSCI, were expressed at very low levels in both spermatocytes and spermatids. Our comparative analysis was then used to identify two X-linked genes that may escape MSCI in spermatocytes, and 21 that are specifically re-activated in spermatids of human, mouse and dog.

**Conclusions:**

Our data indicate that MSCI is incomplete in the dog. This may be partially explained by extensive, but transient, self-synapsis of the X chromosome, in association with rapid completion of meiotic DSB repair. In addition, our comparative analysis identifies novel candidate male fertility genes.

**Electronic supplementary material:**

The online version of this article (doi:10.1186/s12864-015-1501-9) contains supplementary material, which is available to authorized users.

## Background

**Figure 1:**
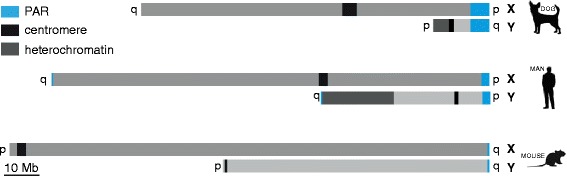
Dog, human, and mouse sex chromosomes. Schematic drawing of the dog, human, and mouse sex chromosomes. The PAR regions are shown in blue. The location of centromeres is indicated by a black box, and the heterochromatic areas on the Y chromosomes are shown in dark gray. p and q arms are indicated.

In placental mammals, the X and Y chromosomes share homology only in the pseudoautosomal regions (PAR). PAR length varies greatly among species, ranging from only 700 kb in mouse [1], to 2.7 Mb (PAR1) in human [2] and 6.6 Mb in dog [3,4] (Figure 1). In the mouse, all chromosomes are acrocentric, and the PAR localizes at the tip of the long q arm of the sex chromosomes. The human metacentric X and Y chromosomes have two PARs, one on each end of the sex chromosomes. However, usually only PAR1, at the end of the p arm of the human sex chromosomes, engages in meiotic recombination [5-8]. The single dog PAR localizes to the tip of the Xp and Yq arms. The total length of the dog Y is only approximately 20 Mb, which is relatively short compared to the human and mouse Y chromosomes with a length of 60 Mb and 95 Mb, respectively. In contrast, the X chromosome is highly conserved in evolution, measuring 156, 171, and 124 Mb in human, mouse, and dog, respectively (Figure 1). The presence of a heterologous chromosome pair in males poses a challenge to male meiosis, and has specific consequences for the transcriptional activity of the sex chromosomes during spermatogenesis as explained below.

In the first meiotic division, homologous chromosomes need to segregate faithfully, to generate two daughter cells that both carry a haploid set of chromosomes. This requires a lengthy prophase during which each chromosome pairs, and exchanges genetic information with its homologous partner. This process involves the formation of approximately 250 meiotic DNA double-strand breaks (DSBs) [9] by the transesterase SPO11 [10,11] at so-called hotspots [12] distributed throughout the genome. Subsequently, repair of these DSBs occurs via a meiosis-specific adaptation of the somatic homologous recombination (HR) repair process. HR in meiotic prophase cells is thought to prefer the use of the intact DNA from the homologous chromosome as a template for repair, whereas the sister chromatid is the favoured repair template in somatic G2 cells [13]. This preference for the homologous chromosome in meiosis is known as the interhomolog bias [14]. Based on this bias, the meiosis-specific HR repair mechanism helps chromosome pairing and allows complete homologous synapsis through the formation of the synaptonemal complex (SC) [15]. The meiotic HR machinery also ensures formation of at least one crossover for each chromosome pair, which is required for faithful segregation of homologous chromosomes and the sex chromosomes during the first meiotic metaphase to anaphase transition. All these events can be traced via immunocytochemical localization of key players in the HR repair process and markers of SC formation. RAD51 and DMC1 are recombinases that form foci in response to the formation of DSBs by SPO11, and each focus is thought to correspond to a DSB site [16,9,17,18]. SC formation reaches completion as DSB repair proceeds. The initial formation of the axial elements, that form first along each chromosomal axis, can be followed by staining for its components, such as SYCP2, SYCP3 [19,20], and the HORMAD proteins [21]. Subsequently, formation of the central or transverse elements, that provide the “glue” between the homologous chromosomes as they synapse, can be followed by staining for components of the transverse filaments and central element such as SYCP1 [22] and TEX12 [23].

Stable XY synapsis in mouse and man is observed only for the tips of these chromosomes that contain the PAR [24]. Detailed electron microscopic evaluation of the XY pair in mouse spermatocytes has revealed that the synapsis between X and Y is dynamic, whereby the region that displays synapsis varies between 0.2 and 3 μm. When synapsis is maximal, approximately 90% of the complete Y-chromosomal axis is synapsed [25,26]. The unsynapsed configuration of the heterologous X and Y chromosomal axes is detected throughout meiotic prophase. Sex chromosomes are thus faced with a particular challenge in repairing meiotic DSBs in the non-PARs. Asynapsis and persistence of unrepaired DSBs lead to global transcriptional repression, a process called meiotic sex chromosome inactivation (MSCI) (reviewed in [27]; [18]). On the other hand, PARs, where synapsis and repair can proceed similarly as on autosomes, are generally thought to escape from MSCI [28], but so far this has not been analysed.

In mouse, inactive XY chromatin forms a distinct nuclear domain, the so-called XY body, which usually resides at the periphery of the nucleus and can be cytologically distinguished by its: 4',6-diamidino-2-phenylindole (DAPI)-intense appearance [29]. XY body formation has been observed in a variety of different mammals [30-38]. Transcriptional suppression of X and Y has been reported to persist in post-meiotic spermatids in mouse and human, and is referred to as post-meiotic sex chromatin repression (PSCR) [39,40]. PSCR, despite being a direct consequence of MSCI, appears to be less stringent, since a significant number of X-linked genes are post-meiotically reactivated (multi-copy genes [41], and ~13% to 20% of single copy genes [39,42]). Transcriptomic studies of MSCI and PSCR have so far been performed only in mouse and human, using microarray analyses of RNA purified from different germ cell types [39,42,43], or whole testes [44]. More recently, also RNA sequencing approaches have been used to analyse global and sex chromosome transcriptional regulation during spermatogenesis in the mouse [45,46]. MSCI appears to be a male-specific specialization of a more general process, meiotic silencing of unsynapsed chromatin (MSUC), that silences chromatin that remains unsynapsed during male and female meiotic prophase in mammals [47,48]. Although MSUC shares several epigenetic characteristics with MSCI, these phenomena seem to lead to opposite end results: MSCI must successfully be installed in order for spermatocytes to progress through the meiotic prophase [49], whereas occurrence of MSUC could be detrimental to the meiotic cells [50]. Whether MSUC and MSCI are equally effective is not known, since no global analyses of mRNA expression from regions that are subject to MSUC are available. Based on immunocytochemical analyses, it appears that some components of a shared MSCI/MSUC machinery are limiting, and when MSUC is activated in spermatocytes, this reduces the effectiveness of MSCI [51]. Meiotic and post-meiotic X chromosome silencing was reported to be similarly effective for mouse and man [43]. However, when we analysed publicly available microarray data from human testes, the results indicated that MSCI and PSCR might be less complete in human compared to mouse [44], and this notion was supported by immunocytological observations that indicated that MSCI was less stringently controlled in human compared to mouse [52]. Furthermore, the profiles of X-linked genes escaping from PSCR diverge significantly between humans and mice [43]. Thus, detailed analysis of MSCI and PSCR in other mammalian species might help in understanding common and relevant features of these processes and identify new important candidate X-linked fertility genes.

In the past, we have studied the functional links between sex chromosome pairing, DSB repair, and transcriptional silencing in several species [53,54,44], and here we have focused on the regulation of the sex chromosomes during male meiotic prophase in the dog (*Canis familiaris*), because of its long PAR (Figure 1), and some peculiar features of XY pairing during meiotic prophase. The last common ancestor of mouse, human and the domestic dog lived around 65 million years ago [55]. The diploid genome of the dog consists of 38 autosomal pairs and the sex chromosomal pair [56]. Previously, the general pattern of meiotic recombination during dog meiotic prophase has been studied through staining for the mismatch repair protein MLH1, which also marks crossover sites in spermatocytes of mouse and man [57].

Herein, we first provide a detailed description of the assembly and disassembly of the synaptonemal complex during canine spermatogenesis. We show that the dog X chromosome displays extensive self-synapsis during mid pachytene, contributing to a more rapid meiotic DSB repair along the self-synapsed part of the X chromosome and to early loss of MSCI marks. These observations, together with the availability of complete high coverage genome sequencing for this species, prompted us to study MSCI and PSCR more in detail by RNA sequencing. We show that X self-synapsis is associated with a partial escape from MSCI. In contrast to what has been previously suggested [28], the PAR, at least in dog, has very low transcriptional activity in both spermatocytes and spermatids. In addition, our comparative analysis among dog, mouse and human of post-meiotically down- and up-regulated genes, provides new potential targets in the search for candidate X-linked male fertility genes.

## Results

### Extensive X self-synapsis in dog mid pachytene spermatocytes

**Figure 2:**
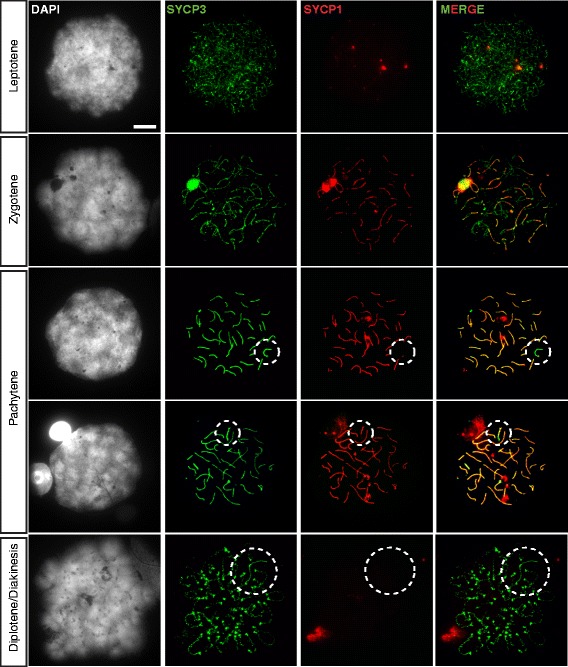
Extensive X chromosome self-synapsis in dog pachytene spermatocytes. Overview of the dog male meiotic prophase and the progression of synapsis between the XY pair. The left panel shows DAPI staining of the different spermatocyte nuclei, the substages are indicated on the left. To the right of the DAPI images, images of the corresponding nuclei stained for the synaptonemal complex proteins SYCP1 (green) and SYCP3 (red) are shown, followed by the merge. The XY pair in pachytene and diplotene nuclei is encircled. Bar represents 10 μm.

**Figure 3:**
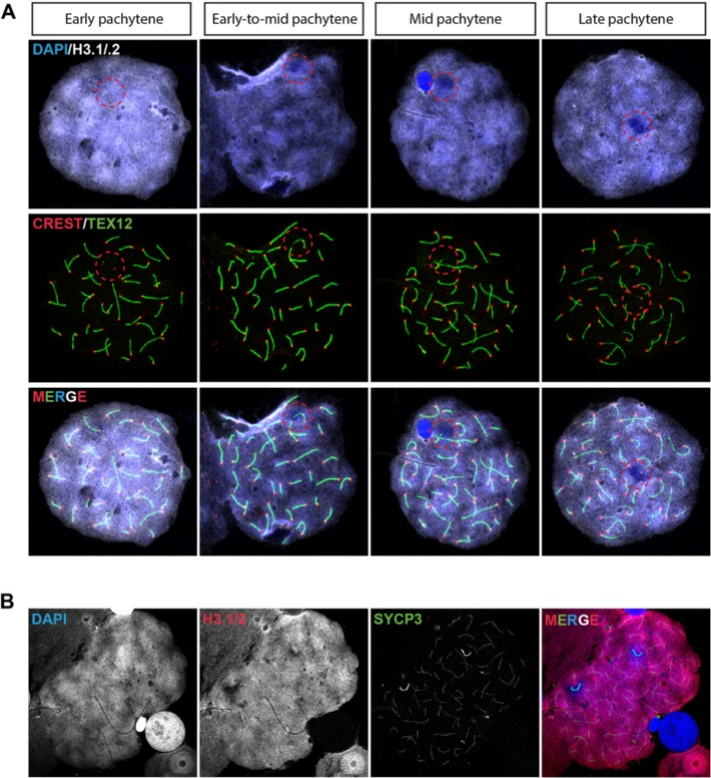
Progressive remodeling of the XY body during meiotic prophase in dog spermatocytes. **A)** Pachytene spermatocyte spread nuclei stained for DAPI (blue), H3.1/2 (white, artificial color chosen to represent the infrared signal), CREST (red) and TEX12 (green). The different substages are indicated on top. **B)** Pachytene spermatocyte spread nucleus stained for DAPI (blue), H3.1/2 (red), SYCP3 (green). Single immunostainings are shown in grayscale.

**Figure 4:**
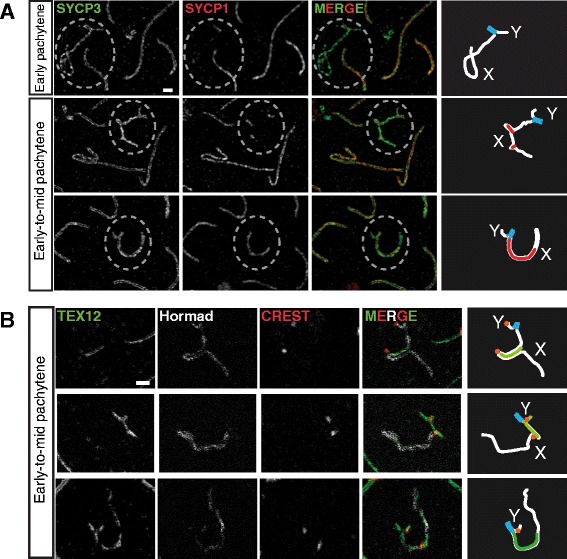
Transient X self-synapsis during pachytene in dog spermatocytes. **A)** Subregions of spermatocyte spread nuclei containing the X and Y at different substages of pachytene indicated on the left are shown at high resolution (Structured Illumination Microscopy (SIM)) to resolve the lateral elements of the synaptonemal complex immunostained for SYCP3 (white/green). The nuclei are costained for SYCP1 to identify regions of synapsis. To the right, drawings showing in white the X-specific and the Y-specific regions, in light blue PAR homologous synapsis and in red X chromosome heterologous self-synapsis. Bar represents 1 μm. **B)** As in A, but these nuclei were stained with antibodies against TEX12 (marker of synapsis), HORMAD1/2 (specifically localizes on unsynapsed axes) and CREST (to mark the centromeres) as indicated. To the right, drawings showing in white the X-specific and the Y-specific region , in light blue PAR homologous synapsis, in green X chromosome heterologous self-synapsis, and in orange the centromere locations. Scale bar represents 1 μm.

The progression of meiotic prophase and synapsis in canine spermatocytes was studied by immunostaining for SYCP3 (lateral elements) and SYCP1 (transverse filaments). At leptotene (2% of the spread nuclei of spermatocytes, n = 100), short fragments of SYCP3 appear throughout the nucleus, some of which become associated with SYCP1 fragments (Figure 2). In zygotene nuclei (13% n = 100), longer stretches of SYCP3 and SYCP1 are present, in agreement with the known progression of synapsis between the homologous chromosomes. However, the SYCP3 pattern appears a bit more dotted compared to what can be observed in mouse zygotene spermatocytes. In pachytene nuclei (81% n = 100), SC assembly was found to be complete on all autosomes, and SYCP3 and SYCP1 completely co-localize, indicating full synapsis. Diplotene nuclei were very rare (4% n = 100), indicating that this stage is very short in the dog (in mouse we observed that 23% of spermatocyte nuclei were at diplotene, n = 100). Again, SYCP3 was observed in a dotted pattern, whereas mouse diplotene spermatocytes still contain linear SYCP3 staining along the chromosomal axes at this stage. The XY pair could be easily detected in the majority of pachytene nuclei, due to the absence of SYCP1 along most of the X chromosomal axis detected by SYCP3 (Figure 2), but no distinct nuclear domain encompassing the XY body was observed in the DAPI staining. The chromosomal axis of the Y chromosome was very short, and frequently appeared to synapse almost completely with the X. In addition, large stretches of SYCP1, much longer than what might have been expected from XY synapsis, were sometimes observed (Figure 2, lower pachytene nucleus). To study the dynamics of XY association during meiotic prophase in more detail, we first identified the different XY configurations, and their temporal appearance. To this end we made use of the fact that XY body formation is followed by a replacement of histone variants H3.1 and H3.2 by H3.3, as has been described for the mouse [58], allowing a distinction between early and mid-to-late pachytene spermatocytes. Using a monoclonal antibody that specifically recognizes H3.1 and H3.2, we observed depletion of the H3.1/2 antigens from at least part of the XY body from mid pachytene onwards (Figure 3A). Costaining with the marker of the central element TEX12, and the centromere marker CREST, revealed that a short synapsed patch can be observed on the XY pair in early pachytene, before histone removal, that localizes near the Y centromere (the dog X and Y chromosomes are metacentric, but since the Y axis is so short, the PAR always appears close to the Y centromere), indicating synapsis between X and Y. Subsequently, when H3.1 removal occurs in early-to-mid pachytene and mid pachytene spermatocytes, a longer stretch of TEX12 staining is observed on the XY pair (Figure 3A). This extensive synapsis is transient, and was usually observed in the area between the PAR and the X chromosome centromere (p-arm). Finally, in late pachytene, the TEX12 signal becomes restricted again to the area close to the Y centromere. At these final stages, the X-chromosomal axis stained by SYCP3 becomes thickened (Figure 3B). When we quantified the extent of self-synapsis along the X chromosome in 100 pachytene nuclei, 27 of these nuclei displayed no detectable XY synapsis (although X and Y were paired), 44 nuclei had little synapsis (either restricted to the PAR or similar to what is shown in the middle panel of Figure 3B), extensive synapsis up to the X centromere was observed in 21 nuclei, and even more extreme synapsis, including part of the Xq region, was detected in 8 nuclei. To understand if the extensive synapsis occurs between X and Y or represents some form of X self-synapsis, we used Structured Illumination Microscopy (SIM) which reaches a resolution of 120 nm (green channel) to 130 nm (red channel), on samples immunostained for SYCP3 and SYCP1 (Figure 4A). This showed a short stretch of synapsis between X and Y, encompassing approximately half of the Y chromosomal axis, in early pachytene nuclei. At mid pachytene, additional synapsis was observed along two short stretches where part of the X chromosome forms a looped structure. Thus, this configuration may represent heterologous self-synapsis of the X. In addition, some nuclei showed a split SYCP3 signal along most of the X chromosome separated by a stretch of SYCP1, indicating that in these nuclei synapsis between sister chromatids of the X chromosome may occur. To verify whether these SYCP1 stretches represented true synapsis, we also stained for TEX12, a component of the central element, and HORMAD1, a marker of unsynapsed axes which is known to be removed upon synapsis in mouse spermatocytes [21]. Figure 4B shows that indeed also in dog spermatocytes HORMAD1 signals are frequently reduced when TEX12 is present. In addition, CREST antibody was used to verify the locations of the X and Y centromeres, and the immunostaining showed that the X centromere localizes at the tip of the loop that is formed and appears to display some form of heterologous synapsis.

### DNA double strand break repair appears to be completed during maximal self-synapsis along the X chromosome axis

**Figure 5:**
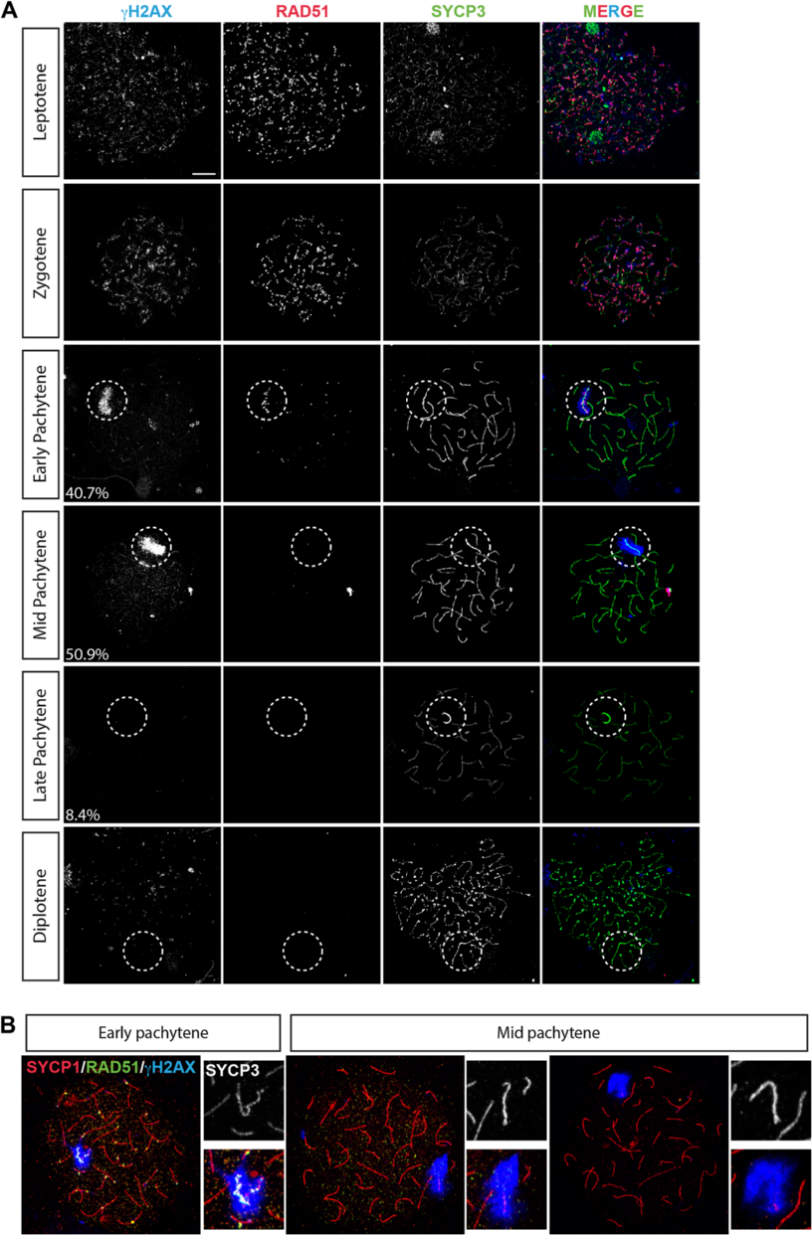
DSBs repair dynamics. **A)** Spermatocyte spread nuclei stained for γH2AX (blue), RAD51 (red) and SYCP3 (green). Single stainings are shown in gray scale. Percentages indicate the fraction of pachytene nuclei observed as shown. Zygotene nuclei were identified based on the fact that axes were split for more than one chromosome pair, and γH2AX and RAD51 staining signals were extensive. Pachytene nuclei were identified based on complete synapsis of the autosomes, and we discriminated between early-pachytene (γH2AX staining of the XY body, and still some RAD51 foci on autosomes), mid-pachytene (γH2AX staining of XY body but no RAD51 foci on autosomes), and late-pachytene (no γH2AX staining on the XY body, and thickened SYCP3 axes of the XY body) spermatocytes. Diplotene nuclei were characterized by desynapsis and thickening of the SYCP3 ends. Scale bar represents 10 μm. **B)** Spermatocyte spread nuclei stained for γH2AX (blue), RAD51 (green), SYCP1 (green), and SYCP3 (only shown in the enlargement, in grayscale). Close-ups show a magnification of the XY body, the top image shows SYCP3 staining (grayscale) in the area, for which the merge of SYCP1, RAD51 and γH2AX is shown below.

Due to the largely heterologous state of the mouse X and Y chromosomes, DSB repair on these sex chromosomes is delayed in regions outside the PAR (reviewed in [59]). To visualize the dynamics of DSB formation and repair on the dog XY, we first analysed the presence of the homologous recombination repair protein RAD51, and the well-known marker of DSBs and the XY body, γH2AX [11] (Figure 5A). γH2AX represents the phosphorylated form of H2AX. This variant of H2A can be phosphorylated by the checkpoint kinases ATR and ATM or by DNA-PKCs in somatic cells in response to DNA damage [60]. During meiotic prophase in mouse, ATM phosphorylates H2AX at meiotic DSBs [61,11,60]. Later, ATR is responsible for the formation of γH2AX on the XY body [61,62].

**Figure 6:**
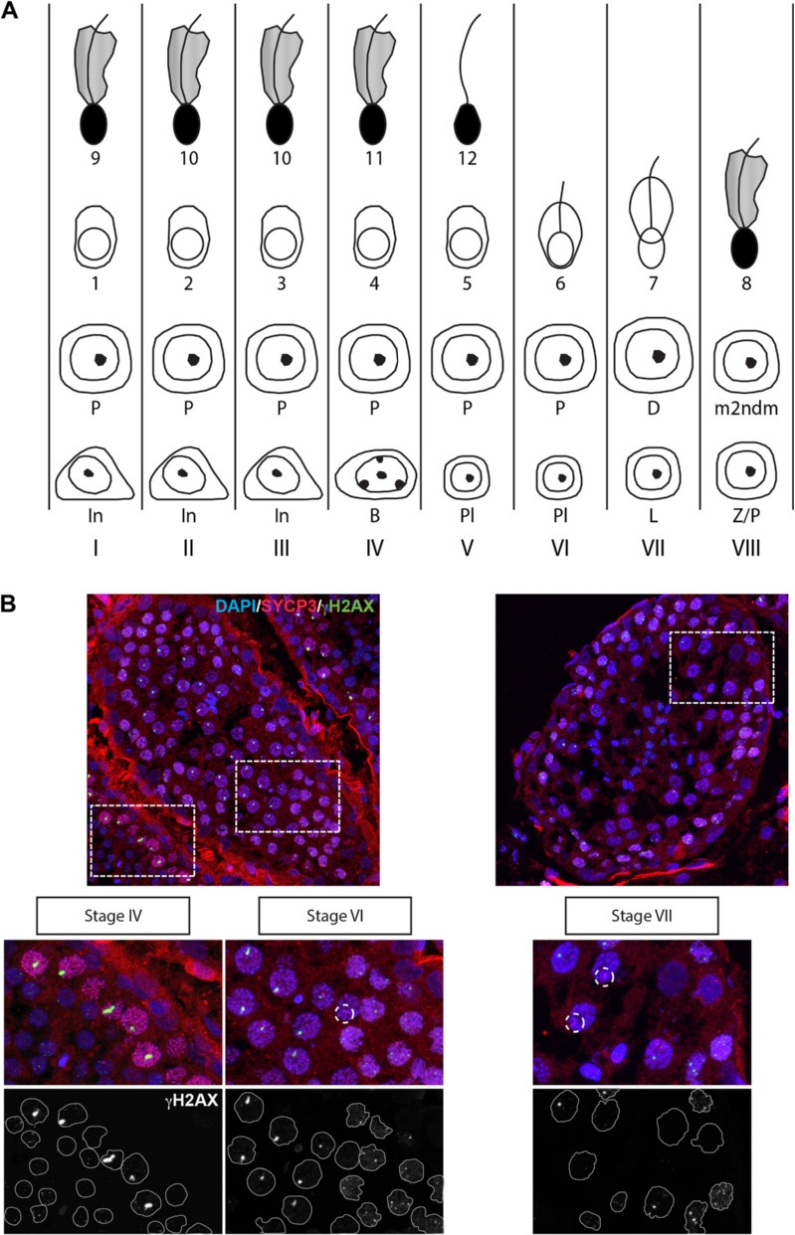
Loss of γH2AX from the XY body in pachytene spermatocytes becomes evident at Stage VI of the spermatogenic cycle in the dog. **A)** Schematic drawing of the different stages of the spermatogenic cycle of the dog (adapted from [71]). **B)** Immunostaining of cryosections of dog testes for γH2AX (green) and SYCP3 (red), and also stained with DAPI (blue). Stages were identified based on Russell et al. (1990) [71]. Loss of γH2AX and the thickened SYCP3 axes representative of late pachytene becomes evident at Stage VI. Single immunostainings are shown in grayscale. Magnifications are shown in the bottom panels, and some cells and the XY body are outlined for reference.

At leptotene, we observed many RAD51 foci, indicative of the presence of DSBs throughout the nucleus, in accordance with a previous report [57]. γH2AX displayed a focal staining pattern colocalising with the RAD51 foci. The number of foci decreased during zygotene. In early pachytene nuclei (which represent 40.7% of the total number of pachytene nuclei (92/226 nuclei)), only a few foci remained present on the synapsed axes of autosomes, whereas the X chromosome still carried many bright RAD51 foci associated with intense γH2AX staining of the XY body chromatin (Figure 5A). In mid pachytene nuclei (50.9% of the total number of pachytene nuclei), γH2AX still covered most of the XY axes, but RAD51 foci had disappeared from the sex chromosomes. Quadruple staining of RAD51, SYCP3, SYCP1, and γH2AX confirmed the notion that RAD51 disappears before γH2AX, and also showed that RAD51 has disappeared when maximal synapsis is reached (Figure 5B). Finally, neither RAD51 foci nor γH2AX were observed on the XY body of late pachytene nuclei (8.4% of the total number of pachytene nuclei), and in none of the few diplotene nuclei that were found. In cells where both γH2AX and RAD51 were absent from the XY body, the SYCP3 axes of the XY pair usually had a thickened appearance, and these were defined as late pachytenes. We verified the absence of γH2AX from such late pachytene nuclei using immunofluorescent staining of dog testis sections. For the spermatogenic cycle of the dog, 8 different cellular associations have been described (schematically depicted in Figure 6A, [63]), with late pachytene and diplotene spermatocytes appearing at Stages VI and VII. Indeed, we observed a few γH2AX negative nuclei with one intense axis staining for SYCP3 at Stage VI, and more such nuclei were observed at Stage VII (Figure 6B).

### Markers of MSCI on the XY body of dog pachytene spermatocytes

In addition to being the earliest known marker of the mouse XY body, γH2AX is required for the initiation of MSCI in mouse spermatocytes [64]. The canine XY pair is highly enriched for γH2AX in early-to-mid pachytene, but loses this mark before exit from pachytene. We wondered if this early depletion of γH2AX from the XY body could be associated with premature release from MSCI. Therefore, we investigated the localization pattern of additional markers of mammalian MSCI.

**Figure 7:**
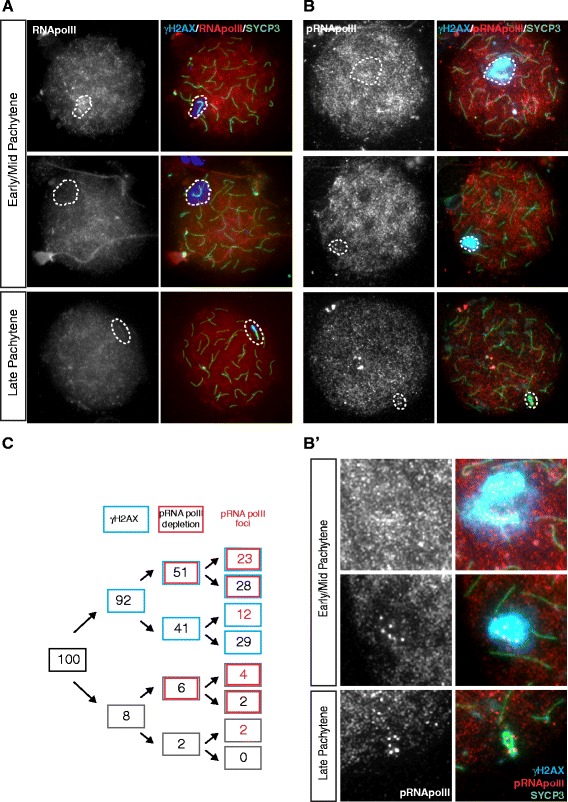
Different patterns of RNA polymerase II localization on the XY body of dog pachytene spermatocytes. **A)** Pachytene spermatocyte spread nuclei stained for γH2AX (blue), RNA polymerase II (RNApolII, red), and SYCP3 (green), single RNA polymerase II immunostainings are shown in gray scale. The lower panel represents a late pachytene spermatocyte in which γH2AX is already lost from part of the X chromosome and RNApolII is not clearly depleted from the XY body. **B)** Pachytene spermatocyte spread nuclei stained for γH2AX (blue), phosphorylated RNA polymerase II (RNApolII, red), and SYCP3 (green), single RNA polymerase II immunostainings are shown in gray scale. The lower panel represents a late pachytene spermatocyte in which γH2AX is already lost from part of the X chromosome. RNA polymerase II depletion from the XY body is evident in the nucleus shown in the middle. In addition, foci of phosphorylated RNA polymerase II staining are specifically observed in the XY body of the middle and the bottom nucleus. **B’** shows magnifications of the XY body regions shown in **B**. **C)** Quantification of the percentages of pachytene nuclei (n = 101) with RNA polymerase II foci in the XY body, relative to the overall depletion of this mark from the (rest of ) the XY body, and to the enrichment of γH2AX in the XY body. Nuclei were immunostained as shown in panel **B**.

**Figure 8:**
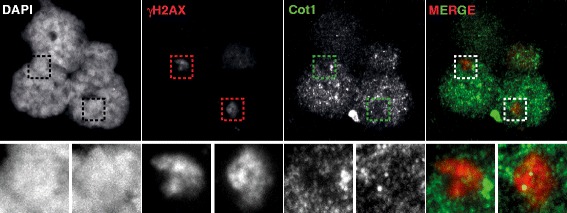
Reduced transcriptional activity in the XY body of dog pachytene spermatocytes. Images of Cot1 RNA FISH combined with an immunostaining for γH2AX on spermatocyte nuclei. High γH2AX signals (covering the XY body) correspond to low Cot1 signals, indicating little transcriptional activity. Magnifications of the left and right XY body are shown below each image.

**Figure 9:**
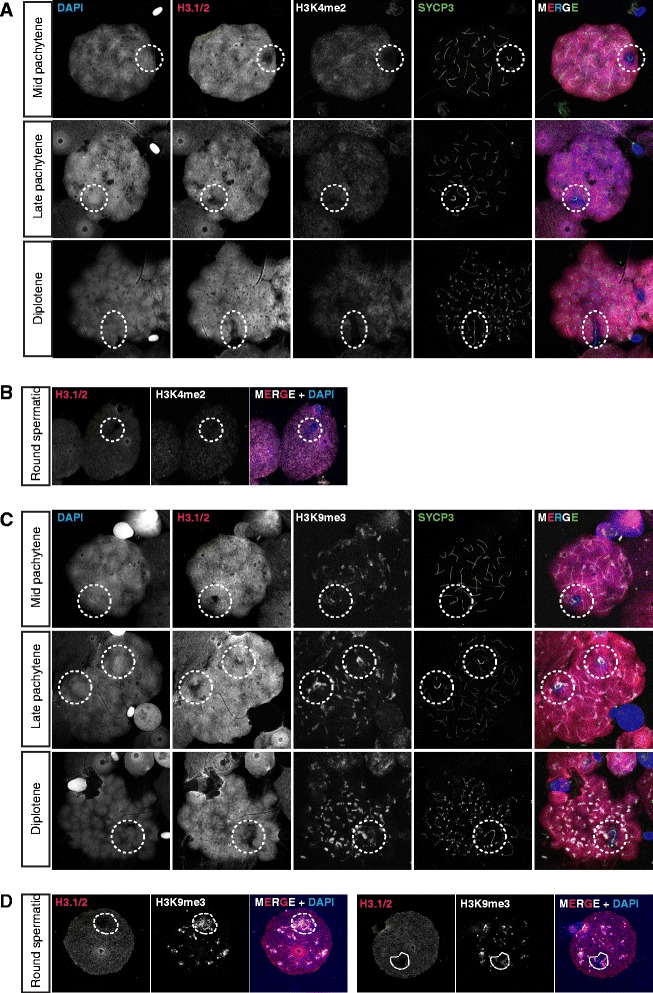
Dynamics of H3K4me2 and H3K9me3 on the XY body in dog spermatocytes. **A)** Pachytene spermatocyte spread nuclei stained for DAPI (blue), H3.1/2 (red), H3K4me2 (white, artificial color chosen to represent the infrared signal) and SYCP3 (green). Single immunostainings are shown in grayscale. Subsequent substages indicated on the left are shown from top to bottom in the different panels. **B)** Spermatid spread nuclei stained for DAPI (blue), H3.1/2 (red), and H3K4me2 (white, artificial color chosen to represent the infrared signal). Single immunostainings are shown in grayscale. **C)** Pachytene spermatocyte spread nuclei stained for DAPI (blue), H3.1/2 (red), H3K9me3 (white, artificial color chosen to represent the infrared signal) and SYCP3 (green). Single immunostainings are shown in grayscale. Subsequent substages indicated on the left are shown from top to bottom in the different panels. **D)** Representative spermatid spread nuclei stained for DAPI (blue), H3.1/2 (red), and H3K9me3 (white, artificial color chosen to represent the infrared signal). Single immunostainings are shown in grayscale.

First, we analysed the presence of RNA polymerase II, using two different antibodies, one targeting all RNA polymerase II, and one that targets only the form that is phosphorylated at serine 2 in the heptapeptide repeats in the C-terminal domain (CTD repeat) of the large subunit in association with transcription elongation (reviewed in [65]). The overall level of RNA polymerase II was found to be quite low in dog pachytene nuclei, but nonetheless the XY pair frequently appeared depleted of RNA polymerase II as compared to the rest of the nucleus (Figure 7A), indicating a lack of transcriptional activity. In accordance with this observation, we also observed reduced staining for phosphorylated RNA polymerase II in the region containing the XY body in the majority of pachytene nuclei (57%, n = 101) (Figure 7B, B’, C). However, surprisingly, this antibody showed an intense dotted signal adjacent to the SYPC3-stained X-chromosomal axis also in a large fraction of the pachytene nuclei (41%), indicating some ongoing RNA transcription along specific regions of the X chromosome. Loss of γH2AX from the XY body was not specifically associated with the appearance of the bright phosphorylated RNA polymerase II foci. Furthermore, when foci were present, the overall level of phosphorylated RNA polymerase II at the XY body was variable (Figure 7C). Thus, at this point, we cannot explain what may cause, and what might be the consequences, of this focal transcriptional activity in the XY body of some pachytene nuclei. To provide additional evidence for overall transcriptional silencing of the XY body, at least in part of the pachytene nuclei, we performed an RNA-FISH experiment to detect nascent transcripts containing repeats. This method has also been used to verify MSCI and MSUC in the mouse [39,47]. Figure 8 indeed shows reduced hybridization of Cot1 DNA in the area of the chromatin that encompasses the XY body in comparison to the rest of the chromatin in two pachytene nuclei (Figure 8). However, the depletion of Cot1 signal was not always evident (not shown). Next, we analysed the localisation of two different histone modifications; H3 lysine 4 dimethylation (H3K4me2), known to be associated with active or potentiated chromatin, and H3 lysine 9 trimethylation (H3K9me3) as a marker of inactive chromatin. In mouse spermatocytes, both these modifications are enriched on the XY body in late prophase nuclei (late pachytene-diplotene) and persist on the sex chromosomes in round spermatids [66,58]. Methylation of H3K9 is thought to function to maintain repression of the sex chromosomes in post-meiotic cells, in the absence of γH2AX [67]. In contrast, the increase in H3K4 methylation in mouse diplotene spermatocytes may contribute to reactivation of a subgroup of X- or Y-linked genes in round spermatids [68]. In dog spermatocytes, the overall level of H3K4me2 was low in pachytene nuclei. In parallel with depletion of H3.1 from the XY body chromatin, depletion of H3K4me2 from XY body chromatin was observed. In diplotene nuclei and round spermatids, H3K4me2 remained excluded from the area of the nucleus that lacks H3.1, which covers either the X or the Y chromosome (Figure 9A and 9B). We performed a similar analysis for H3K9me3, and observed a general enrichment of this modification in the centromeric regions of all chromosomes including the XY pair. Only in late pachytene, enrichment of H3K9me3 on the XY body appeared to spread a bit more, and included at least half of the XY body chromatin, but this enrichment was no longer observed at diplotene (Figure 9C). In round spermatids, enrichment of H3K9me3 was highly variable in the area lacking H3.1, and no specific correlation between lack of H3.1 and increased H3K9me3 signal could be detected (Figure 9D, compare left nucleus to right nucleus).

### mRNA sequencing reveals MSCI and PSCR in the dog

**Figure 10:**
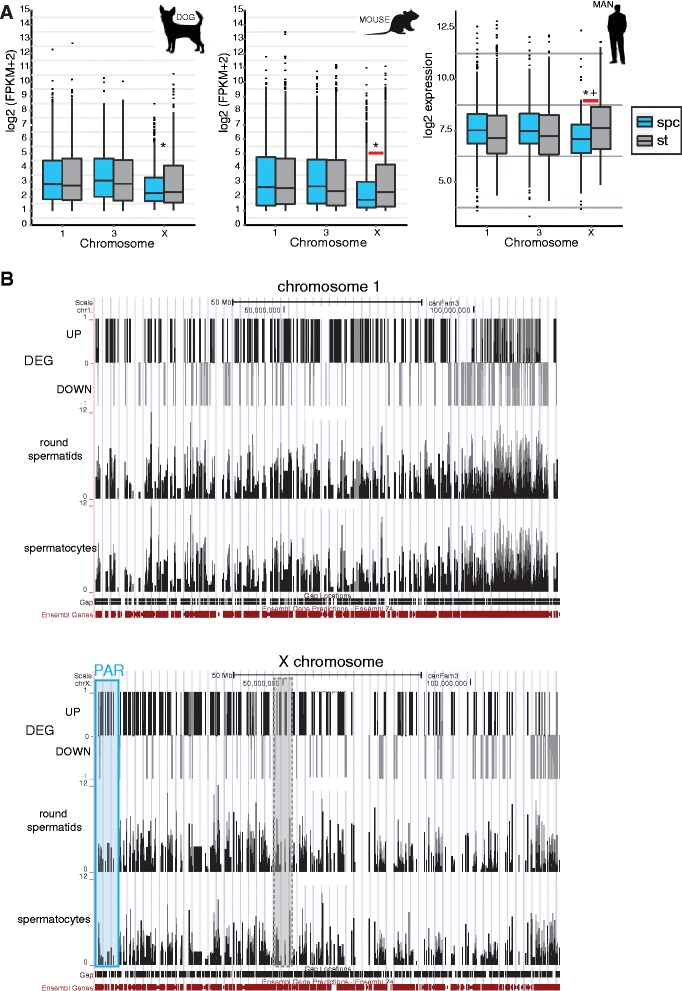
Gene expression in mouse, dog, and human spermatocytes and round spermatids. **A)** Left and middle: boxplots showing median, 25, and 75 percentile log2(FPKM + 2) values of the mRNA levels of genes on chromosomes 1,3, and X in dog and mouse (using the dataset published by [46]) spermatocytes (spc) and round spermatids (st). Genes that were expressed below the 25 percentile values of the whole genome average in both spermatocytes and spermatids were excluded from this analysis. Right: boxplot showing median, 25, and 75 percentile values of the mRNA levels represented as log2(expression value). The values were obtained from published microarray hybridization data using mRNA isolated from human spermatocytes and spermatids [43], and shown here for chromosomes 1,3, and X. Genes with very low expression (value <100, Affimetrix probesets with mean signal intensities <100) in both spermatocytes and spermatids were excluded from this analysis. Asterisks indicate significant difference in gene expression between autosomes and the X chromosome for spermatocytes and spermatids. Plus indicates significantly higher X-linked than autosomal gene expression in spermatids. Horizontal red lines indicate significant difference in X-linked gene expression between spermatocytes and spermatids. **B)** Differentially expressed genes along chromosome 1 and chromosome X, comparing expression in spermatocytes and round spermatids from the dog. Genes that are significantly up-regulated in round spermatids compared to spermatocytes (differentially expressed genes; DEG) are indicated as +1 bars, and significantly down-regulated genes are represented as −1 bars along chromosome X and chromosome 1. In addition, the log2(FPKM + 2) values of all genes along the chromosomes in spermatocytes and round spermatids are shown. Gene density along the chromosomes can be inferred from the density of the bar representing locations of Ensembl annotated genes along the chromosome shown at the bottom. For the X chromosome, the approximate location of the PAR border is indicated by a light blue line, and centromere location is indicated by a dashed gray line.

To directly compare X-chromosomal and autosomal RNA expression levels in both spermatocytes and spermatids, we isolated total RNA from cell preparations enriched in either spermatocytes (79.3% pure, Additional file 1: Table S1) or spermatids (84.4% pure, Additional file 1: Table S1) from testes of 3 dogs and performed RNA sequencing analysis. After removal of genes for which mRNA was absent in 5 or more samples we detected 91% of the genes presently annotated as coding genes in the dog genome (38 autosomes and the X chromosome; the male specific region of the dog Y chromosome sequence was recently reported [4], but has not been annotated). Next, we generated boxplots of the gene expression (log2 FPKM, Fragments Per Kilobase of transcript per Million fragments mapped) and observed that the expression levels for the X chromosome were significantly lower compared to autosomes both in spermatocytes (p-value < 10^−14^) and spermatids (p-value < 10^−5^), indicating that both MSCI and PSCR impact on the activity of the dog X chromosome (Figure 10A, left). To be able to compare the degree of MSCI and PSCR between dog and mouse, we also generated similar boxplots for mouse spermatocytes and spermatids, using the recently published RNA sequencing dataset, using purified spermatocyte and round spermatid cell fractions of similar purity to our samples (Additional file 1: Tables S2 and S3) [46] (Figure 10A, middle). It was found that gene expression from the X compared to the autosomes was reduced more clearly in mouse spermatocytes (p-value <10^−15^). In addition, although PSCR was apparent (p-value < 10^−7^), a significant overall post-meiotic upregulation of X-linked gene expression was observed in mouse (p-value < 10^−15^), but not in dog spermatids. For the human, only results from microarray analyses are available [43], and we generated boxplots of these datasets. The results indicate that X-linked gene expression in human spermatogenesis follows a pattern with measurable MSCI (p-value < 10^−6^), but X-linked gene expression in round spermatids was even higher than autosomal expression (p-value < 0.01) (Figure 10A, right).

### Reactivation of single copy X-linked genes in dog round spermatids, but also post-meiotic downregulation of a subset of X-linked genes

Next we identified differentially expressed genes between spermatocytes and spermatids using two different approaches (Cufflinks (Cuffdiff) and edgeR (see Methods)). Both approaches yielded highly comparable sets of genes (Additional file 1: Table S4: 70–95% identity of significantly up- and down-regulated autosomal and X-linked genes for the two methods), and a more detailed investigation was performed using the Cufflinks analysis results. For X-linked genes with a more than 1.5 fold change in gene expression, we observed that 207 genes were significantly up-regulated in spermatids compared to spermatocytes, while 123 were down-regulated (Table 1). Previously, we have shown that a large group of single-copy genes can escape from PSCR in mouse spermatids [42], contrary to the hypothesis that post-meiotic expression of X-linked genes would require multiple copies of a gene to overcome the repressive marks [41]. Of the up-regulated genes in dog round spermatids, the only known multi-copy genes we could identify that were differentially expressed from the X chromosome were members of the MAGEA and MAGEB gene families, that are also multi-copy in mouse and man. However, the majority of these genes was down-regulated in spermatids compared to spermatocytes. In addition, 32 differentially expressed dog X-linked genes are homologous to multi-copy X-linked genes identified in mouse and human by Mueller et al. [69], but very few of these genes are clearly multi-copy (>2 copies) in dog, and 14 are down-regulated in spermatids compared to spermatocytes (Additional file 1: Table S5).

**Table 1:** **Comparison of the number of differentially expressed genes in mouse and dog spermatocytes and spermatids**

	**# genes analysed**	>1.5 fold change (%)*	Down (%)*	Up (%)*	# X- linked genes (%)*	>1.5 fold change X (%)*	Down X (%)*	Up X (%)*
**Mouse**	23100	8565 (37)	4305 (19)	4260 (18)	1016 (4.4)	399 (1.7)	57 (0.25)	342 (1.5)
**Dog**	19475	8851 (45)	4219 (22)	4632 (24)	768 (3.9)	330 (1.7)	123 (0,63)	207 (1.1)
**Common**	13492	2650 (20)	1340 (10)	1310 (10)	475 (3.5)	100 (0.74)	9 (0.066)	91 (0.67)

Down: down-regulated in spermatids compared to spermatocytes.

Up: up-regulated in spermatids compared to spermatocytes.

*percentage relative to the #genes analysed.

Next we compared the above-mentioned RNA sequencing data recently deposited for mouse spermatocytes and spermatids [46], to our dog dataset. Significantly differentially expressed genes were identified using the same method for both species (shown in Table 1). From this, it is clear that there are more X-linked genes that become up-regulated in spermatids in mouse compared to dog. Conversely, fewer X-linked genes are specifically post-meiotically down-regulated in mouse compared to dog. Almost half of the X-linked genes that are up-regulated in spermatids in the dog share this expression pattern with the mouse. In contrast, less than 10% of the down-regulated genes are shared between mouse and dog.

### Differential regulation of gene expression along the p and q arms of the dog X chromosome

To study whether certain regions of the dog X chromosome (in relation to the observed heterologous self-synapsis) are more likely to be up- or down-regulated in round spermatids we plotted the density of differentially expressed genes and their corresponding expression level (log2 FPKM) along the X chromosome and chromosome 1 (these two chromosomes are similar in size and gene content in dog) (Figure 10B). For the X chromosome, a higher number of up-regulated genes appears to be located on the p arm (containing the PAR) as compared to the q arm. Reciprocally, the q arm of the X chromosome contains a relatively high number of down-regulated genes. For chromosome 1, some areas are also more enriched for either up- or down-regulated genes, but here it appears to correlate better with gene density and the overall gene expression activity in those areas. A similar pattern to that found for chromosome 1 was observed for chromosomes 3 and 5 (data not shown).

**Figure 11:**
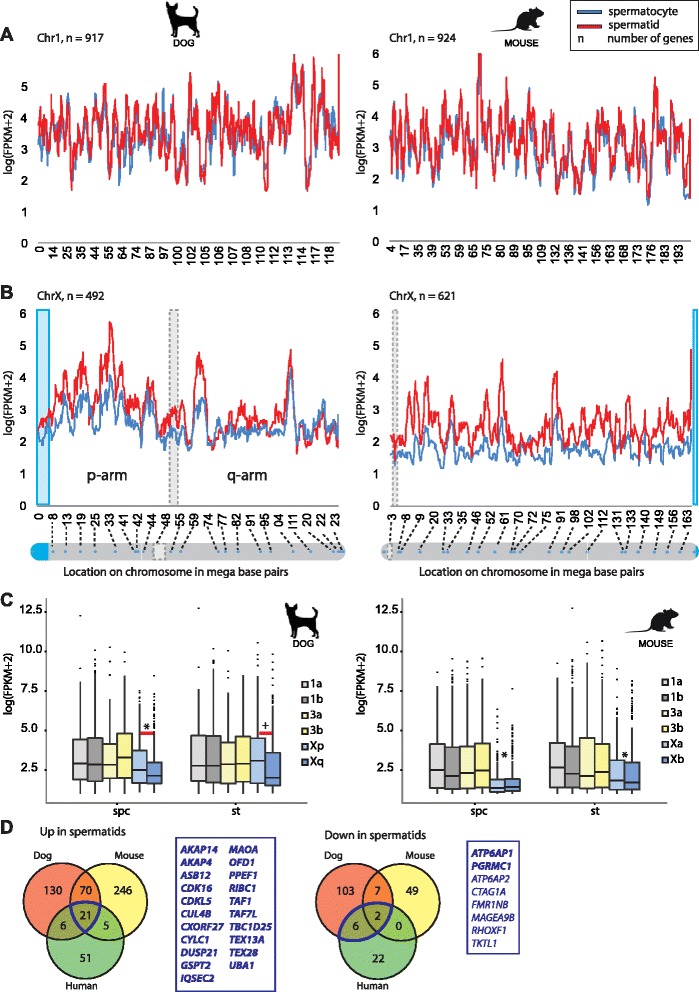
Partial conservation of MSCI and PSCR among mouse, dog, and man. **A)** Walking average of gene expression along chromosome 1 in spermatocytes and round spermatids from dog and mouse. **B)** Walking average of gene expression along chromosome X in spermatocytes and round spermatids from dog and mouse. PARs are indicated in blue. Centromere positions are shown in dashed gray boxes. **C)** Boxplot showing median, 25, and 75 percentile of gene expression in the p arm (Xp) and q arm (Xq) of the dog X in spermatocytes and round spermatids compared to boxplots of arbitrarily split autosomes of dog and mouse X chromosomes and autosomes. Asterisks indicate significant difference in gene expression between split autosomes and the X chromosome arms. Plus indicates significant difference between split autosomes and the q arm of the X only. Horizontal red lines indicate significant difference in X-linked gene expression between chromosome arms. **D)** Venn diagram showing the number of commonly up-regulated (left) and down-regulated (right) X-linked genes in round spermatids compared to spermatocytes in dog, mouse and human. Genes commonly up-regulated or down-regulated in all three species are listed in bold. Down-regulated genes common only to dog and human, not to mouse, are listed with regular characters. Borders of the areas including listed genes are marked in blue.

To further investigate whether changes in gene expression between spermatids and spermatocytes vary along the X chromosome, we plotted the walking average of gene expression along chromosomes X and 1. The expression pattern of genes along chromosome 1 is not globally different between spermatocytes and spermatids (Figure 11A). In contrast, the expression of genes along the X chromosome is globally up-regulated in spermatids compared to spermatocytes (Figure 11B). However, we observed variability in the degree of upregulation depending on the position of genes on the chromosome. Upregulation is much more pronounced along the p arm of the X chromosome, compared to the q arm (Figure 11B). We then looked at the expression level of genes along the X chromosome in mouse, and observed a much more clear upregulation in round spermatids, compared to what we observed for the dog (Figure 11B). Along chromosome 1, the patterns of gene expression were similar in mouse and dog. To visualize the differential regulation of the p and q arms of the X chromosome in the dog more clearly, we generated separate boxplots of gene expression from the two arms in spermatocytes and round spermatids, and compared the outcome to boxplots obtained for autosomes of the dog (Figure 11C). All dog and mouse autosomes, and also the mouse X chromosome, are acrocentric. Therefore, in this evaluation we introduced an arbitrary split of left (a) and right (b) parts of chromosomes 1, 3, and X. As expected, we observed that expression from the dog p and q arm of the X is significantly reduced compared to the split autosomes in spermatocytes (p-value Xp < 0.05, Xq < 10^−7^) (Figure 11C). However, the expression from the p-arm was higher than the expression from the q arm (p-value <0.05). More importantly, in round spermatids, the gene expression from the p arm of the dog X even reaches a level that is not different from autosomal gene expression, whereas the q arm remains repressed (p-value < 10^−3^) (Figure 11C). In the mouse, no differences in gene expression between the arbitrarily split arms of the chromosomes, including the X, were observed (Figure 11C). In accordance with the results presented in Figure 10, partial reactivation is observed for both (arbitrarily chosen) parts of the X chromosome.

### Low gene expression levels in the dog PAR

The dog has an exceptionally large PAR containing 34 annotated genes [56], and it was expected that this region would escape from MSCI because it is capable to fully engage in homologous synapsis and meiotic recombination. However, we found a relatively low average level of mRNA expression in this region (Additional file 1: Table S6) in both spermatocytes and spermatids. The value is more than 10-fold lower than the overall genome average, and also more than two-fold lower than the average of the whole X chromosome in spermatocytes. Thus, it appears that the dog PAR is even more repressed in spermatocytes compared to the rest of the X chromosome, and shows very little re-expression in spermatids. Interestingly, one gene, named *SLC25A6* (*ANC2*), was found to be highly expressed in dog spermatocytes (normalized FPKM = 47.19 in spermatocytes and 18.11 in spermatids), and thus appears to escape from MSCI. We cannot formally exclude that the mRNA molecules that are detected in extracts from the purified spermatocytes were produced in spermatogonia or early spermatocytes, but there are several arguments that indicate that this is highly unlikely. First, the FPKM of 47.19 lies well above the genome average of 27.05 in dog spermatocytes. Second, global gene transcription is very low in leptotene and zygotene spermatocytes [70], and since duration of the pachytene stage is around 13 days in the dog [71], *SLC25A6* mRNA would have to be extremely stable to remain present in the population of purified spermatocytes at such a high level. In ES cells, the median half life of mRNAs is 7.1 h [72], and although regulation of mRNA stability will differ between different cell types, we feel that active transcription of *SLCA25A6* in dog pachytene spermatocytes most likely accounts for the high FPKM value that we measured for this mRNA. *SLC25A6* encodes an adenine nucleotide carrier (ANC), an ADP/ATP carrier that transports ADP into mitochondria and ATP out of mitochondria. SLC25A6 is a member of a family of four proteins that also includes SLC25A4 (ANC1), SLC25A5 (ANC3) and SLC25A31 (ANC4). Of these, only the gene encoding SLC25A5 is also X-linked. The expression levels of these genes in spermatocytes and spermatids of mouse, dog and man are shown in Additional file 1: Table S7. It is clear that both the autosomal *SLC25A31* gene and the X-linked *SLC25A6* gene are the main ANCP variants that are expressed in spermatocytes in dog. *SLC25A6* has not been conserved in the mouse genome, and the autosomal *SLC25A31* variant is highly expressed in spermatocytes. In human spermatocytes, *SLC25A31* is also the main expressed gene, and the two X-linked variants *SLC25A5* and *SLC25A6* are expressed at levels comparable to the median for expressed genes calculated for the genome in both spermatocytes and round spermatids, indicating that they may be transcribed and escape MSCI.

### Pathway analysis of X-linked genes with common post-meiotic regulation between mouse, dog and man

To investigate if genes that are up-regulated in spermatids in both mouse and dog belong to a conserved pathway that is essential during this stage, a pathway analysis was performed using the 91 commonly up-regulated genes in spermatids. The top 5 of significantly enriched Molecular and Cellular Functions are: Cell Morphology; DNA Replication, Recombination, and Repair; Cell Signaling; Post-Translational; and Protein Synthesis (Additional file 1: Table S8). Finally, we compared the X-linked up- and down-regulated genes between dog, mouse, and human (Figure 11D), and identified 21 genes that are up-regulated in spermatids of all three species (Additional file 1: Table S9). For 8 of these genes, mouse knockout models have been described; two are embryonic-lethal, precluding direct analyses of the function of the gene in spermatogenesis, three displayed no clear reproductive defect, and three models displayed defects in post-meiotic spermatid differentiation. For the X-linked genes that were expressed at lower levels in spermatids compared to spermatocytes, very few were conserved between two species, and only two were common to mouse, dog, and man (*ATP6AP1* and *PGRMC1*) (Figure 11D).

## Discussion

Here, we performed a detailed analysis of the dynamics of XY chromosome pairing during meiotic prophase in dog, in relation to the progression of meiotic DSB repair, and regulation of gene expression.

The dog X chromosome has two special characteristics that make this analysis particularly relevant. First, it displays transient but extensive self-synapsis during pachytene, as revealed by our immunocytochemical analyses, and second it has an exceptionally long PAR, as compared to mouse and human. We studied both these properties in relation to the dynamics of meiotic DSB repair and the MSCI and PSCR processes.

### Transient self-synapsis correlates with progression of HR repair on the X chromosome and loss of γH2AX

In general, lack of synapsis is associated with persistence of markers of DSB repair [16]. For the mouse XY pair, this might be attributed to the lack of homology between X and Y, in combination with the effect of an interhomolog bias, which would inhibit use of the sister chromatid as a template for HR repair. The accomplishment of extensive heterologous synapsis along the dog X during a brief period in mid pachytene was associated with concomitant loss of markers of ongoing DSB repair. Loss of γH2AX occurred first in the area that included both the PAR and the self-synapsed regions, occurring either heterologous in looped regions of the X chromosome, or between sister chromatids, indicating that HR repair may occur faster in this area compared to the rest of the X chromosome. This finding also nicely correlates with the fact that we observed loss of HORMAD association with the self-synapsed regions, and HORMAD activity has been clearly linked to ATR activation and subsequent γH2AX formation in the mouse [73,74]. The fact that all γH2AX enrichment on the X is lost prior to the end of pachytene may also indicate that meiotic HR repair on the X is complete before the end of pachytene, and thus may occur overall more rapidly in dog compared to mouse. Although meiotic HR repair is thought to preferentially involve the use of an intact repair template from the homologous chromosome, this is not possible for sex chromosomal regions outside the PAR. In the mouse, it has been suggested that some DSBs might be repaired by non-homologous end-joining at the end of meiotic prophase, when components of this machinery are re-expressed [75]. However, it is perhaps more likely that the sister chromatid is eventually used as a repair template on the sex chromosomes, because the DSB ends have been resected, and this may channel repair towards homologous recombination. In addition, intrachromosomal nonallelic HR (NAHR) may also occur [76]. In this pathway, recombination occurs between repeat sequences with a high sequence homology (usually low copy number repeats) on the same chromosome. This might have deleterious consequences such as inversions or deletions, but if repair is channelled towards a noncrossover pathway, such as synthesis-dependent strand annealing, overall chromosome structure can be retained [76]. Meiotic NAHR has not yet been explored for the dog sex chromosomes. For the human X and Y chromosomes, intrachromosomal NAHR has been reported as a cause of chromosomal aberrations associated with intellectual disability [77] and male infertility [78], respectively. From this, it might be suggested that, at least in the human, intrachromosomal NAHR without genomic rearrangements could also occur during meiotic prophase, and function as a normal pathway to repair (meiotic) DSBs along the unsynapsed axes of X and Y.

### MSCI occurs in dog spermatocytes, but may be incomplete or transient in some regions

Persistence of meiotic DSBs and asynapsis are detected by the machinery that induces MSCI in the mouse [48,47,74,18]. We observed transient extensive self-synapsis of the X and associated loss of γH2AX from synapsed regions in dog pachytene spermatocytes. In addition, although H3K4me2 remains depleted from the X and Y chromosomes in diplotene nuclei, H3K9me3 enrichment on the sex chromosomes was only transiently observed, and not clearly observed in late spermatocytes and post-meiotic cells. Also, in late pachytene and diplotene spermatocytes, RNApolII depletion was less clear compared to the earlier pachytene nuclei, and phosphorylated RNA polymerase was highly enriched in a focal manner along selected regions of the X chromosome in subfraction of the pachytene nuclei, apparently independent of synapsis. This contrasts with what has been observed in mouse, where the depletion of RNApolII on the XY body compared to autosomes is most evident in late pachytene and diplotene, mainly because overall transcription on autosomes increases [70]. Based on these observations, we investigated if MSCI and/or PSCR might be incomplete, or transient in the dog, using a whole genome approach.

Using RNA sequencing, we observed that MSCI occurs also in the dog. When we analysed global regulation of gene expression along the dog X chromosome in spermatocytes and round spermatids, we observed that MSCI is less clearly established along the p arm of the X chromosome, compared to the q arm. In addition, the overall level of gene expression derived from the p arm of the X chromosome was similar to that of autosomal genes in round spermatids, whereas for the q arm post-meiotic reactivation of the X-linked genes was limited or even absent. It is interesting to note that the X self-synapsis is most frequently observed along the p arm of the X chromosome, and synapsis of the tip of the q arm was almost never observed. Thus, it might be suggested that the (heterologous) synapsis facilitates not only DSB repair, but also helps to (re)activate gene expression on the X chromosome. Still, there are also regions in the q arm of the X chromosome that appear to have a relatively high expression level in both spermatocytes and round spermatids. Intriguingly, the very region for which we would expect a clear correlation between synapsis and lack of MSCI, the PAR, was expressed at very low levels in both spermatocytes and spermatids. We expected that the PAR would behave like autosomes regarding repair and synapsis, and escape from MSCI, but it appears to be subject to its own unknown specific regulatory control mechanism. Thus, although there is some degree of correlation between X self-synapsis and reduced MSCI, other, yet unknown aspects of sex chromosome regulation may differ between dog and mouse, and influence MSCI.

### MSCI escapees

Little is known about X-linked genes that may escape from MSCI in mouse, except for a report on escape of miRNA genes and the noncoding *Tsx* transcript in spermatocytes [79,80].

Since MSCI is less complete in dog (and possibly also in man [44,52], and observations in this manuscript), it might be expected that true escapees of MSCI could exist in these species. In dog, 46 of the X-linked genes that are more than 1.5-fold down-regulated in spermatids have an FPKM value >4 in spermatocytes, which is higher than the median FPKM value of all genes that were analysed in spermatocytes, and thus indicates ongoing transcription.

X-linked genes that are specifically expressed in spermatocytes and become down-regulated in spermatids in more than one species are expected to possibly exert a relevant function during meiosis. Mouse and dog have 9 common X-linked genes that are expressed at a level more than 1.5-fold lower in spermatids compared to spermatocytes. Two of these, *ATP6AP1/Atp6ap1* and *PGRMC1/Pgrmc1* are also more than 1.5 fold down-regulated in human spermatids, based on the microarray data. Nothing is known about the putative expression or role of these proteins in spermatocytes.

Interestingly, of the 8 X-linked genes that are down-regulated both in dog and human round spermatids, 5 have been shown to be expressed in human spermatocytes and thus may represent true escapees of MSCI: *FMR1NB*, *CTAG1A* [81], *MAGEA* [82], *RHOXF1* [83], and *TKTL1* [84]. Out of these, FMR1NB, CTAG1A and MAGEA9B are cancer/testis antigens. RHOXF1 is a homeobox protein and TKTL1 is a transketolase involved in the pentose-phosphate pathway. These 5 genes are thus of special interest as candidate X-linked genes that may perform an important function during meiotic prophase in human spermatogenesis. For the other three genes, formal proof of MSCI escape may be obtained when their mRNA expression levels in purified spermatogonia can be directly compared to their mRNA levels in purified spermatocytes.

The gene encoding the ADP/ATP carrier SLC25A6 is another interesting putative MSCI-escapee, and localizes to the PAR in dog and man. ANC proteins are of vital importance for the metabolism of the cell, and pachytene spermatocytes likely require a high rate of ATP synthesis [85,86].

### PSCR may not reflect an active repressive process

From our whole genome analysis of mRNA expression in dog spermatocytes and spermatids, it is clear that most X-linked genes that are significantly up-regulated in spermatids compared to spermatocytes are single-copy genes. We propose that persistence of the inactivation of X-linked genes in dog and perhaps human spermatids is not primarily due to a post-meiotic repressive mechanism operating during this stage, but rather represents a carry-over of MSCI. This is supported by the observed lack of accumulation of H3K9me3, as well as by the absence of a DAPI dense area on the sex chromosomes in spermatids, one of the markers of PSCR in mouse. This new extensive analysis of MSCI and PSCR in the dog again supports the notion that X-linked gene activation occurs frequently for single-copy genes in spermatids, and is not globally inhibited. It should also be considered that expression of X-linked genes in spermatids will not only require alleviation of MSCI but also more specific post-meiotic transcriptional regulation.

### Identification of putative X-linked male fertility genes

The pathway analysis of X-linked genes that are significantly up-regulated in spermatids both in mouse and dog, revealed that most of these genes are involved in shaping the sperm cell and maintaining genome integrity. These are important processes that require (re)activation of a specific subset of X-linked genes. In addition, it is worth mentioning that a relatively high number of reactivated genes are involved in the regulation of posttranslational modifications and of protein synthesis and degradation (in particular the ubiquitin pathway). The addition of the dog as a new species to the dataset of gene expression in spermatocytes and spermatids allows identification of X-linked genes that show post-meiotic induction of gene expression in mouse, human and dog, thus providing more evidence for an important function in spermatogenesis. For some of these genes, knockout mice have been generated. Several of these mutant animals were shown to be fertile, indicating that even conserved expression does not necessarily indicate that the gene is absolutely required for fertility, at least in the mouse. It should be kept in mind that spermatogenesis is much more efficient in mouse compared to man, yielding a much higher number of mature spermatozoa per gram testis weight per time unit, and a small selective advantage of expression of an X-linked gene in mouse spermatids may translate into an essential function in man. Therefore, all genes listed in Additional file 1: Table S9, as well as the conserved possible MSCI escapees described above should be considered candidate human fertility genes.

## Conclusions

Our results provide novel insights in the regulation and significance of sex chromosome gene expression during spermatogenesis in mammals. We show that transient but extensive heterologous self-synapsis of the X chromosome is associated with rapid DSB repair and reduced MSCI. On the other hand, the genes that are localized in the pseudoautosomal region are mostly transcriptionally inactive in both spermatocytes and spermatids, despite the normal progression of synapsis in this region. We hypothesize that the PAR is controlled by its own specific regulatory mechanism but that specific genes in this region that exert a meiotic or post-meiotic function can be active. Furthermore, based on conserved expression patterns in mouse, human, and dog spermatocytes and/or spermatids, we identified genes that may function in spermatocytes (escapees) or round spermatids (reactivated genes), and should be considered as potential X-linked male fertility factors.

## Methods

### Sample collection and germ cell isolation

Testes used in this study were not obtained in the context of animal experiments, but collected as remnant material from specialized veterinary clinics immediately after dog castration surgery was performed using the standard procedure (adhering to best practice of veterinary care) of that clinic, at the owners’ request, and not for the purpose of the experiment. No aspect of the timing of the operation, the operation procedure itself, or further treatment or health perspective of the animals, was influenced by the collection of the testes. Testis material was first collected in normal saline solution, transported on ice, and further processed as described below for different applications. For RNA sequencing analysis, testes were obtained from two Chihuahuas, here named dog 4 and dog 6 (respectively 9 and 16 months old), and from one German Shepherd, referred to as dog 5 (4 years old). Spermatocytes and round spermatids were purified on the day of testes collection by using collagenase and trypsin treatment, followed by sedimentation at unit gravity (StaPut procedure) [87].

### Analyses of cell purity in the isolated germ cell fractions

Purified germ cells were fixed in Bouins' fixative on glass slides and stained with eosin and hematoxylin using standard histological methods. Purity was estimated by cellular morphology (percentages are shown in Additional file 1: Table S1). In addition, we compared the RNA-seq transcript levels of five genes specifically expressed in spermatids (three protamine genes [*PRM1, PRM2*, and *PRM3*] and two transition protein genes [*TNP1* and *TNP2*]) and 3 genes that are specifically induced in spermatocytes (*SYCP1, 2,* and *3*), between the spermatid and spermatocyte samples, and between the dog and (published) mouse samples (Additional file 1: Tables S8 and S9). From this, it is clear that the fold changes in gene expression are similar in the mouse and dog samples, indicating comparable purities. This is also consistent with the reported purities of these mouse spermatocyte and round spermatid fractions (approximately 70 and 90%, repectively, based on morphological assessment [46]). Since the spermatid-specific mRNAs are not expressed in spermatocytes, the average log2 of the fold-change (FC) of 3.6 of increased expression in the spermatid fraction, indicates a contamination of around 8% of spermatid mRNA in the spermatocyte mRNA preparations. This is consistent with the results of the morphological assessment. For contamination of spermatocytes in the spermatid fraction, no such calculation can be made, since RNA transcription of genes that are important in spermatocytes may continue to some extent in round spermatids.

### Antibodies

For primary antibodies, we used: mouse monoclonal antibodies anti-phosphorylated H2AX (Upstate), anti-RNA polymerase II (Abcam), anti-Ser2 phosphoylated RNA Polymerase II (H5) (Abcam) anti H3.1/2 (gift from dr. P. de Boer); rabbit polyclonal antibodies anti-RAD51 [88], anti-SYCP3 and anti-SYCP1 (gift from dr. C. Heyting), anti-HORMAD1 (gift from dr. A. Tóth), anti-H3K4me2, anti-H3K9me3; goat anti-SYCP3 (R&D System); guinea pig anti-TEX12 (gift from dr. C. Höög); human anti-centromere antibodies (CREST) (Fitzgerald Industries). For secondary antibodies, we used a goat anti-rabbit IgG alexa 405/488/546/633, goat anti-mouse alexa IgG 350/488/546/633, donkey anti-goat IgG alexa 488/555, goat-anti human IgG 555, goat anti-guinea pig IgG 488/546 (Molecular Probes).

### Cryosections

Parts of the testes were fixed (4% v/v paraformaldehyde in PBS) on ice for 4 hours. Samples were immersed in 30% w/v sucrose (in water) overnight and subsequently embedded in OCT (Optimal Cutting Temperature compound). Crysections of 10 μm were used for immunostaining as described below.

### Meiotic spread nuclei preparations and immunocytochemistry

Testis tissues were either directly processed to obtain spread nuclei for immunocytochemistry, or first snap frozen and processed upon thawing in PBS, as described in [89]. Spread nuclei of spermatocytes were stained with antibodies mentioned above. Before incubation with antibodies, slides were washed in phosphate buffered saline (PBS, 3 × 10 min), and non-specific sites were blocked with 0.5% w/v BSA and 0.5% w/v milk powder in PBS. Primary antibodies were diluted in 10% w/v BSA in PBS, and incubations were overnight at room temperature in a humid chamber. Subsequently, slides were washed (3 × 10 min) in PBS, blocked in 10% v/v normal goat serum (Sigma) in blocking buffer (supernatant of 5% w/v milk powder in PBS centrifuged at 14,000 rpm for 10 min), and incubated with secondary antibodies in 10% normal goat serum in blocking buffer at room temperature for 2 hours. Finally, slides were washed (3 × 10 min) in PBS (in the dark) and embedded in Prolong Gold with or without DAPI (Invitrogen).

### Imaging

Fluorescent images were observed using a fluorescence microscope (Axioplan 2; Carl Zeiss) equipped with a digital camera (Coolsnap-Pro; Photometrics). Fluorescent images were taken under identical conditions for all slides, and images were analyzed using the ImageJ (Fiji) software (Rasband, W.S., ImageJ, U.S. National Institutes of Health, Bethesda, Maryland, USA [http://rsb.info.nih.gov/ij/]). Confocal imaging was performed on a Zeiss LSM700 microscope (Carl Zeiss, Jena): we used a 63× oil immersion objective lens (N.A. 1.4), pinhole 1 AU. DAPI was excited at 405 nm and imaged with a short pass filter (SP) 490 nm; Alexa 488 was excited at 490 nm and imaged SP 555 nm; Alexa 546 was excited at 555 nm and imaged SP 640 nm; Alexa 633 was excited at 639 nm and for the imaging no filter was required. SIM analysis was performed using a Nikon N-SIM super-resolution microscope system and NIS-Elements 2 image processing software.

### RNA fluorescent *in situ* hybridization (FISH)

We carried out Cot-1 RNA FISH using a previously described method [47]. Dog Cot-1 DNA was prepared from dog genomic DNA by shearing, denaturing, and reannealing under conditions that enrich for repetitive sequences [90].

### RNA sequencing

Total RNA was extracted from (pure) spermatocyte and spermatid fractions using the Trizol RNA isolation protocol. Quality of extracted RNA was verified using Bioanalyzer 2100 (Agilent technologies), all samples had RIN values above 8.5. RNA sequencing library was prepared using Illumina TruSeq RNA version 1 according to the manufacturer’s protocol starting with 1 μg of total RNA. RNA sequencing was performed on Illumina HiSeq2000 for single read 36 bp. Sequence reads (2.1-4.6 million reads per sample, of which between 91-93% could be aligned) were aligned to the dog genome (*Canis familiaris*,NCBI, build3.1) using Tophat (version Tophat-1.4.0). Transcripts were assembled and expression values (FPKM) were generated using Cufflinks (version Cufflinks-2.1.1). Data was further processed to calculate gene expression per chromosome using R and Excel. First, genes with low data and FPKM less than 0 in more than 5 samples out of 6 were removed. To remove low expressed genes, we first calculated the 25 percentile of all samples, and removed genes with FPKM below the 25 percentile in spermatocytes.

Differential expression was performed using Cufflinks (Cuffdiff) [91] and edgeR [92]. For Cuffdiff, Cufflinks-assembled transcripts were first merged using Cuffmerge, and differentially expressed genes were identified using Cuffdiff. Cuffdiff results were further processed using cummeRbund. For edgeR, reads per gene were first counted using HTSeq [http://www-huber.embl.de/users/anders/HTSeq/] and differential expression was assessed between the two groups, each with three replicates. Pathway analysis (enrichment analysis) was performed using IPA (Ingenuity® Systems, www.ingenuity.com). Statistical analysis (Wilcoxon rank sum test) was performed using R software.

## Additional file

Additional file 1: Table S1. Analyses of dog germ cell purity. **Table S2.** Relative expression levels of spermatocyte genes in mouse and dog spermatids. **Table S3.** Relative expression levels of spermatid genes in mouse and dog spermatids. **Table S4.** Comparison between Cufflinks and edgeR results. **Table S5.** Differentially expressed dog X-linked genes homologous to mouse and/or human multicopy genes. **Table S6.** Average FPKM values of gene expression in dog. **Table S7.** Expression of *SLC25A* variants in spermatocytes and spermatids of mouse man and dog. **Table S8.** Molecular and cellular functions associated with X-linked genes upregulated in spermatids of dog and mouse. **Table S9.** X-linked genes upregulated in spermatids of mouse, human, and dog.

## Abbreviations

ANC: Adenine nucleotide carrier
DAPI: 4',6-diamidino-2-phenylindole
DSBs: DNA double-strand breaks
FC: Fold-change
FISH: Fluorescent in situ hybridization
FPKM: Fragments Per Kilobase of transcript per Million fragments mapped
HR: Homologous recombination
MSCI: Meiotic Sex Chromosome Inactivation
MSUC: Meiotic Silencing of Unsynapsed Chromatin
NAHR: Nonallelic homologous recombination
PAR: Pseudoautosomal region
PBS: Phosphate buffered saline
PSCR: Postmeiotic Sex Chromatin Repression
SC: Synaptonemal complex
SIM: Structured illumination microscopy

## References

[1] Perry J, Palmer S, Gabriel A, Ashworth A (2001). A short pseudoautosomal region in laboratory mice. Genome Res.

[2] Ross MT, Grafham DV, Coffey AJ, Scherer S, McLay K, Muzny D (2005). The DNA sequence of the human X chromosome. Nature.

[3] Young AC, Kirkness EF, Breen M (2008). Tackling the characterization of canine chromosomal breakpoints with an integrated in-situ/in-silico approach: the canine PAR and PAB. Chromosome Res.

[4] Li G, Davis BW, Raudsepp T, Pearks Wilkerson AJ, Mason VC, Ferguson-Smith M (2013). Comparative analysis of mammalian Y chromosomes illuminates ancestral structure and lineage-specific evolution. Genome Res.

[5] Henke A, Fischer C, Rappold GA (1993). Genetic map of the human pseudoautosomal region reveals a high rate of recombination in female meiosis at the Xp telomere. Genomics.

[6] Hinch AG, Altemose N, Noor N, Donnelly P, Myers SR (2014). Recombination in the human Pseudoautosomal region PAR1. PLoS Genet.

[7] Page DC, Bieker K, Brown LG, Hinton S, Leppert M, Lalouel JM (1987). Linkage, physical mapping, and DNA sequence analysis of pseudoautosomal loci on the human X and Y chromosomes. Genomics.

[8] Rouyer F, Simmler MC, Johnsson C, Vergnaud G, Cooke HJ, Weissenbach J (1986). A gradient of sex linkage in the pseudoautosomal region of the human sex chromosomes. Nature.

[9] Moens PB, Kolas NK, Tarsounas M, Marcon E, Cohen PE, Spyropoulos B (2002). The time course and chromosomal localization of recombination-related proteins at meiosis in the mouse are compatible with models that can resolve the early DNA-DNA interactions without reciprocal recombination. J Cell Sci.

[10] Keeney S, Baudat F, Angeles M, Zhou ZH, Copeland NG, Jenkins NA (1999). A mouse homolog of the Saccharomyces cerevisiae meiotic recombination DNA transesterase Spo11p. Genomics.

[11] Mahadevaiah SK, Turner JM, Baudat F, Rogakou EP, de Boer P, Blanco-Rodriguez J (2001). Recombinational DNA double-strand breaks in mice precede synapsis. Nat Genet.

[12] Smagulova F, Gregoretti IV, Brick K, Khil P, Camerini-Otero RD, Petukhova GV (2011). Genome-wide analysis reveals novel molecular features of mouse recombination hotspots. Nature.

[13] Schwacha A, Kleckner N (1997). Interhomolog bias during meiotic recombination: meiotic functions promote a highly differentiated interhomolog-only pathway. Cell.

[14] Bolcun-Filas E, Schimenti JC (2012). Genetics of meiosis and recombination in mice. Int Rev Cell Mol Biol.

[15] Yang F, Wang PJ (2009). The Mammalian synaptonemal complex: a scaffold and beyond. Genome Dyn.

[16] Moens PB, Chen DJ, Shen Z, Kolas N, Tarsounas M, Heng HHQ (1997). Rad51 immunocytology in rat and mouse spermatocytes and oocytes. Chromosoma.

[17] Cole F, Kauppi L, Lange J, Roig I, Wang R, Keeney S (2012). Homeostatic control of recombination is implemented progressively in mouse meiosis. Nat Cell Biol.

[18] Carofiglio F, Inagaki A, de Vries S, Wassenaar E, Schoenmakers S, Vermeulen C (2013). SPO11-independent DNA repair foci and their role in meiotic silencing. PLoS Genet.

[19] Lammers JH, Offenberg HH, van Aalderen M, Vink AC, Dietrich AJ, Heyting C (1994). The gene encoding a major component of the lateral elements of synaptonemal complexes of the rat is related to X-linked lymphocyte-regulated genes. Mol Cell Biol.

[20] Offenberg HH, Schalk JA, Meuwissen RL, van Aalderen M, Kester HA, Dietrich AJ (1998). SCP2: a major protein component of the axial elements of synaptonemal complexes of the rat. Nucleic Acids Res.

[21] Wojtasz L, Daniel K, Roig I, Bolcun-Filas E, Xu H, Boonsanay V (2009). Mouse HORMAD1 and HORMAD2, two conserved meiotic chromosomal proteins, are depleted from synapsed chromosome axes with the help of TRIP13 AAA-ATPase. PLoS Genet.

[22] Meuwissen RL, Offenberg HH, Dietrich AJ, Riesewijk A, van Iersel M, Heyting C (1992). A coiled-coil related protein specific for synapsed regions of meiotic prophase chromosomes. EMBO J.

[23] Hamer G, Gell K, Kouznetsova A, Novak I, Benavente R, Hoog C (2006). Characterization of a novel meiosis-specific protein within the central element of the synaptonemal complex. J Cell Sci.

[24] Solari AJ (1974). The behavior of the XY pair in mammals. Int Rev Cytol.

[25] Solari AJ (1970). The spatial relationship of the X and Y chromosomes during meiotic prophase in mouse spermatocytes. Chromosoma.

[26] Tres LL (1977). Extensive pairing of the XY bivalent in mouse spermatocytes as visualized by whole-mount electron microscopy. J Cell Sci.

[27] Turner JM (2007). Meiotic sex chromosome inactivation. Development.

[28] McKee BD, Handel MA (1993). Sex chromosomes, recombination, and chromatin conformation. Chromosoma.

[29] Monesi V (1965). Differential rate of ribonucleic acid synthesis in the autosomes and sex chromosomes during male meiosis in the mouse. Chromosoma.

[30] Namekawa SH, VandeBerg JL, McCarrey JR, Lee JT (2007). Sex chromosome silencing in the marsupial male germ line. Proc Natl Acad Sci U S A.

[31] Franco MJ, Sciurano RB, Solari AJ (2007). Protein immunolocalization supports the presence of identical mechanisms of XY body formation in eutherians and marsupials. Chromosome Res.

[32] Echeverria OM, Benavente R, Ortiz R, Vazquez-Nin GH (2003). Ultrastructural and immunocytochemical analysis of the XY body in rat and Guinea pig. Eur J Histochem.

[33] Solari AJ, Pigozzi MI (1994). Fine structure of the XY body in the XY1Y2 trivalent of the bat Artibeus lituratus. Chromosome Res.

[34] Villagomez DA (1993). Zygotene-pachytene substaging and synaptonemal complex karyotyping of boar spermatocytes. Hereditas.

[35] Ansari HA, Jung HR, Hediger R, Fries R, Konig H, Stranzinger G (1993). A balanced autosomal reciprocal translocation in an azoospermic bull. Cytogenet Cell Genet.

[36] Libbus BL (1985). The ordered arrangement of chromosomes in the Chinese hamster spermatocyte nucleus. Hum Genet.

[37] Moses MJ (1977). Synaptonemal complex karyotyping in spermatocytes of the Chinese hamster (Cricetulus griseus). II. Morphology of the XY pair in spread preparations. Chromosoma.

[38] Sciurano RB, Rahn MI, Rossi L, Luaces JP, Merani MS, Solari AJ (2012). Synapsis, recombination, and chromatin remodeling in the XY body of armadillos. Chromosome Res.

[39] Namekawa SH, Park PJ, Zhang LF, Shima JE, McCarrey JR, Griswold MD (2006). Postmeiotic sex chromatin in the male germline of mice. Curr Biol.

[40] Turner JM, Mahadevaiah SK, Ellis PJ, Mitchell MJ, Burgoyne PS (2006). Pachytene asynapsis drives meiotic sex chromosome inactivation and leads to substantial postmeiotic repression in spermatids. Dev Cell.

[41] Mueller JL, Mahadevaiah SK, Park PJ, Warburton PE, Page DC, Turner JM (2008). The mouse X chromosome is enriched for multicopy testis genes showing postmeiotic expression. Nat Genet.

[42] Mulugeta Achame E, Wassenaar E, Hoogerbrugge JW, Sleddens-Linkels E, Ooms M, Sun ZW (2010). The ubiquitin-conjugating enzyme HR6B is required for maintenance of X chromosome silencing in mouse spermatocytes and spermatids. BMC Genomics.

[43] Sin HS, Ichijima Y, Koh E, Namiki M, Namekawa SH (2012). Human postmeiotic sex chromatin and its impact on sex chromosome evolution. Genome Res.

[44] Mulugeta Achame E, Baarends WM, Gribnau J, Grootegoed JA (2010). Evaluating the relationship between spermatogenic silencing of the X chromosome and evolution of the Y chromosome in chimpanzee and human. PLoS One.

[45] Lesch BJ, Dokshin GA, Young RA, McCarrey JR, Page DC (2013). A set of genes critical to development is epigenetically poised in mouse germ cells from fetal stages through completion of meiosis. Proc Natl Acad Sci U S A.

[46] Soumillon M, Necsulea A, Weier M, Brawand D, Zhang X, Gu H (2013). Cellular source and mechanisms of high transcriptome complexity in the mammalian testis. Cell Rep.

[47] Turner JM, Mahadevaiah SK, Fernandez-Capetillo O, Nussenzweig A, Xu X, Deng CX (2005). Silencing of unsynapsed meiotic chromosomes in the mouse. Nat Genet.

[48] Baarends WM, Wassenaar E, van der Laan R, Hoogerbrugge JW, Sleddens-Linkels E, Hoeijmakers JH (2005). Silencing of unpaired chromatin and histone H2A ubiquitination in mammalian meiosis. Mol Cell Biol.

[49] Royo H, Polikiewicz G, Mahadevaiah SK, Prosser H, Mitchell M, Bradley A (2010). Evidence that meiotic sex chromosome inactivation is essential for male fertility. Curr Biol.

[50] Burgoyne PS, Mahadevaiah SK, Turner JM (2009). The consequences of asynapsis for mammalian meiosis. Nat Rev Genet.

[51] Homolka D, Ivanek R, Capkova J, Jansa P, Forejt J (2007). Chromosomal rearrangement interferes with meiotic X chromosome inactivation. Genome Res.

[52] de Vries M, Vosters S, Merkx G, D'Hauwers K, Wansink DG, Ramos L (2012). Human male meiotic sex chromosome inactivation. PLoS One.

[53] Schoenmakers S, Wassenaar E, Hoogerbrugge JW, Laven JS, Grootegoed JA, Baarends WM (2009). Female meiotic sex chromosome inactivation in chicken. PLoS Genet.

[54] Schoenmakers S, Wassenaar E, Laven JS, Grootegoed JA, Baarends WM (2010). Meiotic silencing and fragmentation of the male germline restricted chromosome in zebra finch. Chromosoma.

[55] O'Leary MA, Bloch JI, Flynn JJ, Gaudin TJ, Giallombardo A, Giannini NP (2013). The placental mammal ancestor and the post-K-Pg radiation of placentals. Science.

[56] Lindblad-Toh K, Wade CM, Mikkelsen TS, Karlsson EK, Jaffe DB, Kamal M (2005). Genome sequence, comparative analysis and haplotype structure of the domestic dog. Nature.

[57] Basheva EA, Bidau CJ, Borodin PM (2008). General pattern of meiotic recombination in male dogs estimated by MLH1 and RAD51 immunolocalization. Chromosome Res.

[58] van der Heijden GW, Derijck AA, Posfai E, Giele M, Pelczar P, Ramos L (2007). Chromosome-wide nucleosome replacement and H3.3 incorporation during mammalian meiotic sex chromosome inactivation. Nat Genet.

[59] Inagaki A, Schoenmakers S, Baarends WM (2010). DNA double strand break repair, chromosome synapsis and transcriptional silencing in meiosis. Epigenetics.

[60] Rogakou EP, Boon C, Redon C, Bonner WM (1999). Megabase chromatin domains involved in DNA double-strand breaks in vivo. J Cell Biol.

[61] Bellani MA, Romanienko PJ, Cairatti DA, Camerini-Otero RD (2005). SPO11 is required for sex-body formation, and Spo11 heterozygosity rescues the prophase arrest of Atm−/− spermatocytes. J Cell Sci.

[62] Royo H, Prosser H, Ruzankina Y, Mahadevaiah SK, Cloutier JM, Baumann M (2013). ATR acts stage specifically to regulate multiple aspects of mammalian meiotic silencing. Genes Dev.

[63] Foote RH, Swierstra EE, Hunt WL (1972). Spermatogenesis in the dog. Anat Rec.

[64] Fernandez-Capetillo O, Mahadevaiah SK, Celeste A, Romanienko PJ, Camerini-Otero RD, Bonner WM (2003). H2AX is required for chromatin remodeling and inactivation of sex chromosomes in male mouse meiosis. Dev Cell.

[65] Hsin JP, Manley JL (2012). The RNA polymerase II CTD coordinates transcription and RNA processing. Genes Dev.

[66] Khalil AM, Boyar FZ, Driscoll DJ (2004). Dynamic histone modifications mark sex chromosome inactivation and reactivation during mammalian spermatogenesis. Proc Natl Acad Sci U S A.

[67] Cocquet J, Ellis PJ, Yamauchi Y, Mahadevaiah SK, Affara NA, Ward MA (2009). The multicopy gene Sly represses the sex chromosomes in the male mouse germline after meiosis. PLoS Biol.

[68] Baarends WM, Wassenaar E, Hoogerbrugge JW, Schoenmakers S, Sun ZW, Grootegoed JA (2007). Increased phosphorylation and dimethylation of XY body histones in the Hr6b-knockout mouse is associated with derepression of the X chromosome. J Cell Sci.

[69] Mueller JL, Skaletsky H, Brown LG, Zaghlul S, Rock S, Graves T (2013). Independent specialization of the human and mouse X chromosomes for the male germ line. Nat Genet.

[70] Page J, de la Fuente R, Manterola M, Parra MT, Viera A, Berrios S (2012). Inactivation or non-reactivation: what accounts better for the silence of sex chromosomes during mammalian male meiosis?. Chromosoma.

[71] Russell LD, Ettlin RA, Sinha Hikim AP, Clegg ED (1990). Histological and histopathological evaluation of the testis.

[72] Sharova LV, Sharov AA, Nedorezov T, Piao Y, Shaik N, Ko MS (2009). Database for mRNA half-life of 19 977 genes obtained by DNA microarray analysis of pluripotent and differentiating mouse embryonic stem cells. DNA Res.

[73] Daniel K, Lange J, Hached K, Fu J, Anastassiadis K, Roig I (2011). Meiotic homologue alignment and its quality surveillance are controlled by mouse HORMAD1. Nat Cell Biol.

[74] Wojtasz L, Cloutier JM, Baumann M, Daniel K, Varga J, Fu J (2012). Meiotic DNA double-strand breaks and chromosome asynapsis in mice are monitored by distinct HORMAD2-independent and -dependent mechanisms. Genes Dev.

[75] Ahmed EA, Philippens ME, Kal HB, de Rooij DG, de Boer P (2010). Genetic probing of homologous recombination and non-homologous end joining during meiotic prophase in irradiated mouse spermatocytes. Mutat Res.

[76] Sasaki M, Lange J, Keeney S (2010). Genome destabilization by homologous recombination in the germ line. Nat Rev Mol Cell Biol.

[77] Froyen G, Belet S, Martinez F, Santos-Reboucas CB, Declercq M, Verbeeck J (2012). Copy-number gains of HUWE1 due to replication- and recombination-based rearrangements. Am J Hum Genet.

[78] Lange J, Noordam MJ, van Daalen SK, Skaletsky H, Clark BA, Macville MV (2013). Intrachromosomal homologous recombination between inverted amplicons on opposing Y-chromosome arms. Genomics.

[79] Song R, Ro S, Michaels JD, Park C, McCarrey JR, Yan W (2009). Many X-linked microRNAs escape meiotic sex chromosome inactivation. Nat Genet.

[80] Anguera MC, Ma W, Clift D, Namekawa S, Kelleher RJ, Lee JT (2011). Tsx produces a long noncoding RNA and has general functions in the germline, stem cells, and brain. PLoS Genet.

[81] von Kopylow K, Kirchhoff C, Jezek D, Schulze W, Feig C, Primig M (2010). Screening for biomarkers of spermatogonia within the human testis: a whole genome approach. Hum Reprod.

[82] Chen YT, Chiu R, Lee P, Beneck D, Jin B, Old LJ (2011). Chromosome X-encoded cancer/testis antigens show distinctive expression patterns in developing gonads and in testicular seminoma. Hum Reprod.

[83] Song HW, Anderson RA, Bayne RA, Gromoll J, Shimasaki S, Chang RJ (2013). The RHOX homeobox gene cluster is selectively expressed in human oocytes and male germ cells. Hum Reprod.

[84] Rolland AD, Lavigne R, Dauly C, Calvel P, Kervarrec C, Freour T (2013). Identification of genital tract markers in the human seminal plasma using an integrative genomics approach. Hum Reprod.

[85] Brower JV, Lim CH, Jorgensen M, Oh SP, Terada N (2009). Adenine nucleotide translocase 4 deficiency leads to early meiotic arrest of murine male germ cells. Reproduction.

[86] Nakada K, Sato A, Yoshida K, Morita T, Tanaka H, Inoue S (2006). Mitochondria-related male infertility. Proc Natl Acad Sci U S A.

[87] Grootegoed JA, Jansen R, van der Molen HJ (1986). Effect of glucose on ATP dephosphorylation in rat spermatids. J Reprod Fertil.

[88] Essers J, Hendriks RW, Wesoly J, Beerens CE, Smit B, Hoeijmakers JH (2002). Analysis of mouse Rad54 expression and its implications for homologous recombination. DNA Repair (Amst).

[89] Peters AH, Plug AW, van Vugt MJ, de Boer P (1997). A drying-down technique for the spreading of mammalian meiocytes from the male and female germline. Chromosome Res.

[90] Zwick MS, Hanson RE, Islam-Faridi MN, Stelly DM, Wing RA, Price HJ (1997). A rapid procedure for the isolation of C0t-1 DNA from plants. Genome.

[91] Trapnell C, Williams BA, Pertea G, Mortazavi A, Kwan G, van Baren MJ (2010). Transcript assembly and quantification by RNA-Seq reveals unannotated transcripts and isoform switching during cell differentiation. Nat Biotechnol.

[92] Robinson MD, McCarthy DJ, Smyth GK (2010). edgeR: a Bioconductor package for differential expression analysis of digital gene expression data. Bioinformatics.

