# Supplementary material for: Incomplete meiotic sex chromosome inactivation in the domestic dog
Source: BMC Genomics. 2015 Apr 12;16(1):291. doi: 10.1186/s12864-015-1501-9 (PMC4399420; doi:10.1186/s12864-015-1501-9)
Supplement: Additional file 1: Table S1. — Analyses of dog germ cell purity. Table S2. Relative expression levels of spermatocyte genes in mouse and dog spermatids. Table S3. Relative expression levels of spermatid genes in mouse and dog spermatids. Table S4. Comparison between Cufflinks and edgeR results. Table S5. Differentially expressed dog X-linked genes homologous to mouse and/or human multicopy genes. Table S6. Average FPKM values of gene expression in dog. Table S7. Expression of SLC25A variants in spermatocytes and spermatids of mouse man and dog. Table S8. Molecular and cellular functions associated with X-linked genes upregulated in spermatids of dog and mouse. Table S9. X-linked genes upregulated in spermatids of mouse, human, and dog. [file 12864_2015_1501_MOESM1_ESM.pdf]

Table S1: Analyses of dog germ cell purity

	**Spermatocytes**	Spermatids	Cytoplasm	Others	**Spermatids**	Spermatocytes	Cytoplasm	Others
Dog 4	86	16	2	3	140	2	17	6
Dog5	95	18	4	9	101	1	12	8
Dog6	94	9	1	7	116	1	15	4
Tot.	265 **(79.3%)**	43 (12.9%)	7 (2.1%)	19 (5.7%)	357 **(84.4%)**	4 (0.9%)	44 (10.4%)	18 (4.3%)

Table S2: Relative expression levels of spermatocyte genes in mouse and dog spermatids

Spermatocyte gene	Dog log2 fold change spt/spc	Mouse log2 fold change spt/spc
Sycp1/SYCP1	-0.46	-0.42
Sycp2/SYCP2	-2.88	-1.38
Sycp3/SYCP3	-1.07	-1.11
Average	-1.47	-0.97

Table S3: Relative expression levels of spermatid genes in mouse and dog spermatids

Spermatid gene	Dog log2 fold change spt/spc	Mouse log2 fold change spt/spc
Tnp1/TNP1	3.76	3.54
Tnp2/TNP2	4.02	3.51
Prm1/PRM1	n.a.	3.51
Prm2/PRM2	3.86	3.80
Prm3/PRM3	2.75	3.18
Average	3.60	3.51

Table S4: Comparison between Cufflinks and edgeR results

	# diff. expressed genes	# in common (%)	# sign and >1.5 fold up	# in common (%)	# sign and >1.5 fold down	# in common (%)	# sign and >1.5 fold up X	# in common (%)	#sign and >1.5 fold down X	# in common (%)
Cufflinks	9679	8661 (89)	4632	4323 (94)	4220	4094 (97)	207	197 (95)	126	116 (92)
edgeR	9904	8661 (87)	4828	4323 (90)	4919	4094 (83)	224	197 (88)	160	116 (73)

Table S5: Differentially expressed dog X-linked genes homologous to mouse and/or human multicopy genes

**Down in spermatids**	**Up in spermatids**
DDX26B	*COL4A6*
MAGEB1	*GLRA2*
MAGEB4	*GLRA4*
MAGED1	*H2BFWT*
MAOB	*MAGEB10*
NGFRAP1	*MAGEB5*
RAB9A	*MAOA*
SHROOM2	*MID2*
SLC9A6	*PHKA2*
SSX5	*PRPS2*
TCEAL4	*RAB9B*
ZNF182	*SCML1*
ZNF185	*SCML2*
ZNF280C	*SPACA5*
	TGIF2LX
	ZCCHC12
	ZNF711
	ZNF81

Table S6: Average FPKM values of gene expression in dog

	Genome	Chr 1	X	PAR
SPERMATOCYTES	27.05	28.31	6.15	2.46
SPERMATIDS	37.92	35.18	22.87	2.71

Table S7: Expression of *SLC25A* variants in spermatocytes and spermatids of mouse man and dog

DOG (RNA-seq)				MOUSE (RNA-seq)			MAN (microarray)		
gene	chr	spc (FPKM)	spt (FPKM)	chr	spc (FPKM)	spt (FPKM)	chr	spc	spt
SLC25A4	16	5.77	2.06	8	6.18	2.81	4	26.44	13.83
SLC25A5	X	4.08	1.77	X	8.50	2.38	X	159.86	297.73
SLC25A6	X(PAR)	47.19	18.11	-	-	-	X(PAR)	113.95	163.43
SLC25A31	19	34.76	20.54	3	34.10	16.26	4	265.19	75.93

Table S8: Molecular and cellular functions associated with X-linked genes upregulated in spermatids of dog and mouse

Category	p-value	Genes
Cell Morphology	9.44E-04-4.66E-02	*PHEX,AKAP4,PAK3,CDK16,TAF7L,MAOA,HMGB3*
DNA Replication, Recombination, and Repair	2.08E-03-3.94E-02	*HUWE1,CUL4B,CETN2,UBE2A,TEX11,ERCC6L*
Cell Signaling	2.37E-03-4.33E-02	*PRKX,TAF1,AKAP4,PDK3,MED12,MED14*
Post-Translational Modification	2.37E-03-3.17E-02	*HUWE1,CDKL5,OGT,PRKX,TAF1,UBE2A,RLIM,UBA1,ASB9,PDK3,MAOA*
Protein Synthesis	2.37E-03-3.56E-02	*CUL4B,PRKX,TAF1,RLIM,EIF1AX,ASB9,PDK3*
Molecular Transport	3.49E-03-1.99E-02	*PHEX,SLC35A2,RPGR,MAOA*
Nucleic Acid Metabolism	3.49E-03-1.99E-02	*CTPS2,SLC35A2,RPGR*
Small Molecule Biochemistry	3.49E-03-4.33E-02	*OGT,CTPS2,SLC35A2,RPGR,SMS,MAOA*
Carbohydrate Metabolism	4.02E-03-2.39E-02	*OGT,SLC35A2*
Cell Death and Survival	4.02E-03-4.98E-02	*HUWE1,OGT,UXT,ATP11C,PAK3,BGN,SH3KBP1,MAOA*
Cell-To-Cell Signaling and Interaction	4.02E-03-4.71E-02	*CDKL5,OGT,OPHN1,PAK3,RLIM,BGN,MAOA*
Cellular Assembly and Organization	4.02E-03-3.94E-02	*OPHN1,PAK3,OFD1,TEX11,MAOA*
Cellular Development	4.02E-03-4.71E-02	*CUL4B,RLIM,BGN,USP9X,CDK16,ZFX,TEX11,TAF7L*
Cellular Function and Maintenance	4.02E-03-1.99E-02	*PHEX,OPHN1,OFD1,MAOA*
Cellular Growth and Proliferation	4.02E-03-4.33E-02	*CUL4B,CETN2,RLIM,BGN*
Drug Metabolism	4.02E-03-2.39E-02	*MAOA*
Gene Expression	4.02E-03-1.72E-02	*MED12,MED14,TXLNG*
Cell Cycle	8.01E-03-3.17E-02	*RLIM,ERCC6L*
Cellular Movement	8.01E-03-4.11E-02	*CDKL5,AKAP4,TAF7L,MAOA*
Energy Production	8.01E-03-1.6E-02	*MAOA*
Lipid Metabolism	1.6E-02-1.6E-02	*SLC35A2*
Protein Degradation	3.06E-02-3.06E-02	*CUL4B,RLIM,ASB9*
Amino Acid Metabolism	3.17E-02-3.17E-02	*SMS*
RNA Post-Transcriptional Modification	4.71E-02-4.71E-02	*CSTF2*

Table S9: X-linked genes upregulated in spermatids of mouse, human, and dog

	**Gene**	**Mouse knockout**	**Molecular function**	**Putative function in spermatogenesis**
Structural components	*AKAP14*	-	A-kinase-anchoring protein	Ciliary beat frequency [1]
	AKAP4	immotile sperm [2]	A-kinase-anchoring protein	Fibrous sheet formation
	CDK16	malformed sperm , reduced motility [3]	Cyclin dependent kinase	Annulus formation
	OFD1	embryonic lethal [4] Mutations in the human ortholog are associated with Oral–facial–digital type I (OFDI; MIM 311200) syndrome and Simpson-Golabi-Behmel syndrome type 2	Centrosome associated proteins, involved in Cilia formation [5]. Removal through autophagy is essential for proper cilia function [6]	
	CYLC1	-	Cyclicin, basic protein, structural component of cytoskeleton	Component of sperm head cytoskeleton [7]
Ubiquitin pathway	*ASB12*	-	The ankyrin repeat and SOCS box (ASB) family, forms E3 complex with Cul5-Rbx2	Ubiquitylation [8]
	CUL4B	embryonic lethal, epiblast-specific knockout is viable, reproduction not analysed [9]	Component of E3 ligase complex	Ubiquitylation
	UBA1	-	Ubiquitin-activating enzyme	Ubiquitylation
Mitochondrial enzymes	*DUSP21*	-	Mitochondrial membrane phosphatase [10]	
	MAOA	fertile, aggressive behaviour [11]	Monoamine oxidase A degrades serotonin and norepinephrine. Mitochondrial enzyme	
Transcription regulation	*TAF1*	-	Largest subunit of TFIID, transcriptional regulation	
	TAF7L	reduced sperm numbers and quality, fertile [12]	Regulation of transcription program together with TAF1	
	IQSEC2	human mutations cause nonsyndromic intellectual disability [13]	Guanine nucleotide exchange factor for the ADP-ribosylation factor family of small GTPases, involved in membrane trafficking and actin dynamics	
	TBC1D25	-	Rab GTPase-activating protein involved in autophagy [14]	
	CDKL5	neurodevelopmental disorder, fertile [15]	Cyclin-dependent kinase-like	
	CXORF27	-	Huntingtin interacting protein with histone fold [16]	

	GSPT2	-	Polypeptide release factor (translation termination) [17]	
	TEX13A	-	Testis-specific gene [18] possibly involved in mRNA processing [19]	
	PPEF1	fertile, no clear phenotype [20]	Calcium binding phosphatase	
	RIBC1	-	The RIB43A domain with coiled coils 1 (*RIBC1*) gene with unknown function	
	TEX28	-	Testis-specific single exon gene of unknown function [21]	

References

1. Kultgen PL, Byrd SK, Ostrowski LE, Milgram SL. Characterization of an A-kinase anchoring protein in human ciliary axonemes. Mol Biol Cell. 2002;13:4156-66.
2. Miki K, Willis WD, Brown PR, Goulding EH, Fulcher KD, Eddy EM. Targeted disruption of the Akap4 gene causes defects in sperm flagellum and motility. Dev Biol. 2002;248:331-42.
3. Mikolcevic P, Rainer J, Geley S. Orphan kinases turn eccentric: a new class of cyclin Y-activated, membrane-targeted CDKs. Cell Cycle. 2012;11:3758-68.
4. Ferrante MI, Zullo A, Barra A, Bimonte S, Messaddeq N, Studer M et al. Oral-facial-digital type I protein is required for primary cilia formation and left-right axis specification. Nat Genet. 2006;38:112-7.
5. D'Angelo A, De Angelis A, Avallone B, Piscopo I, Tammaro R, Studer M et al. Ofd1 controls dorso-ventral patterning and axoneme elongation during embryonic brain development. PLoS One. 2012;7:e52937.
6. Tang Z, Lin MG, Stowe TR, Chen S, Zhu M, Stearns T et al. Autophagy promotes primary ciliogenesis by removing OFD1 from centriolar satellites. Nature. 2013;502:254-7.
7. Hess H, Heid H, Franke WW. Molecular characterization of mammalian cylicin, a basic protein of the sperm head cytoskeleton. J Cell Biol. 1993;122:1043-52.
8. Kohroki J, Nishiyama T, Nakamura T, Masuho Y. ASB proteins interact with Cullin5 and Rbx2 to form E3 ubiquitin ligase complexes. FEBS Lett. 2005;579:6796-802.
9. Liu L, Yin Y, Li Y, Prevedel L, Lacy EH, Ma L et al. Essential role of the CUL4B ubiquitin ligase in extra-embryonic tissue development during mouse embryogenesis. Cell Res. 2012;22:1258-69.
10. Rardin MJ, Wiley SE, Murphy AN, Pagliarini DJ, Dixon JE. Dual specificity phosphatases 18 and 21 target to opposing sides of the mitochondrial inner membrane. J Biol Chem. 2008;283:15440-50.

11. Cases O, Seif I, Grimsby J, Gaspar P, Chen K, Pournin S et al. Aggressive behavior and altered amounts of brain serotonin and norepinephrine in mice lacking MAOA. Science. 1995;268:1763-6.
12. Cheng Y, Buffone MG, Kouadio M, Goodheart M, Page DC, Gerton GL et al. Abnormal sperm in mice lacking the Taf7l gene. Mol Cell Biol. 2007;27:2582-9.
13. Shoubridge C, Walikonis RS, Gecz J, Harvey RJ. Subtle functional defects in the Arf-specific guanine nucleotide exchange factor IQSEC2 cause non-syndromic X-linked intellectual disability. Small GTPases. 2010;1:98-103.
14. Popovic D, Akutsu M, Novak I, Harper JW, Behrends C, Dikic I. Rab GTPase-activating proteins in autophagy: regulation of endocytic and autophagy pathways by direct binding to human ATG8 modifiers. Mol Cell Biol. 2012;32:1733-44.
15. Wang IT, Allen M, Goffin D, Zhu X, Fairless AH, Brodkin ES et al. Loss of CDKL5 disrupts kinome profile and event-related potentials leading to autistic-like phenotypes in mice. Proc Natl Acad Sci U S A. 2012;109:21516-21.
16. Marino-Ramirez L, Hsu B, Baxevanis AD, Landsman D. The Histone Database: a comprehensive resource for histones and histone fold-containing proteins. Proteins. 2006;62:838-42.
17. Le Goff C, Zemlyanko O, Moskalenko S, Berkova N, Inge-Vechtomov S, Philippe M et al. Mouse GSPT2, but not GSPT1, can substitute for yeast eRF3 in vivo. Genes Cells. 2002;7:1043-57.
18. Wang PJ, McCarrey JR, Yang F, Page DC. An abundance of X-linked genes expressed in spermatogonia. Nat Genet. 2001;27:422-6.
19. Nguyen CD, Mansfield RE, Leung W, Vaz PM, Loughlin FE, Grant RP et al. Characterization of a family of RanBP2-type zinc fingers that can recognize single-stranded RNA. J Mol Biol. 2011;407:273-83.
20. Ramulu P, Kennedy M, Xiong WH, Williams J, Cowan M, Blesh D et al. Normal light response, photoreceptor integrity, and rhodopsin dephosphorylation in mice lacking both protein phosphatases with EF hands (PPEF-1 and PPEF-2). Mol Cell Biol. 2001;21:8605-14.
21. Hanna MC, Platts JT, Kirkness EF. Identification of a gene within the tandem array of red and green color pigment genes. Genomics. 1997;43:384-6.
